# Generation and Characterization of Induced Pluripotent Stem Cells from Aid-Deficient Mice

**DOI:** 10.1371/journal.pone.0094735

**Published:** 2014-04-09

**Authors:** Ren Shimamoto, Naoki Amano, Tomoko Ichisaka, Akira Watanabe, Shinya Yamanaka, Keisuke Okita

**Affiliations:** 1 Center for iPS Cell Research and Application (CiRA), Kyoto University, Kyoto, Japan; 2 Gladstone Institute of Cardiovascular Disease, San Francisco, California, United States of America; University of Kansas Medical Center, United States of America

## Abstract

It has been shown that DNA demethylation plays a pivotal role in the generation of induced pluripotent stem (iPS) cells. However, the underlying mechanism of this action is still unclear. Previous reports indicated that activation-induced cytidine deaminase (Aid, also known as Aicda) is involved in DNA demethylation in several developmental processes, as well as cell fusion-mediated reprogramming. Based on these reports, we hypothesized that Aid may be involved in the DNA demethylation that occurs during the generation of iPS cells. In this study, we examined the function of Aid in iPS cell generation using Aid knockout (*Aid* ^−/−^) mice expressing a GFP reporter under the control of a pluripotent stem cell marker, Nanog. By introducing Oct3/4, Sox2, Klf4 and c-Myc, Nanog-GFP-positive iPS cells could be generated from the fibroblasts and primary B cells of *Aid* ^−/−^ mice. Their induction efficiency was similar to that of wild-type (*Aid* ^+/+^) iPS cells. The *Aid* ^−/−^ iPS cells showed normal proliferation and gave rise to chimeras, indicating their capacity for self-renewal and pluripotency. A comprehensive DNA methylation analysis showed only a few differences between *Aid* ^+/+^ and *Aid* ^−/−^ iPS cells. These data suggest that Aid does not have crucial functions in DNA demethylation during iPS cell generation.

## Introduction

Induced pluripotent stem (iPS) cells can be generated by introducing defined factors into somatic cells [1]. iPS cells have the capacity for self-renewal and pluripotency, similar to embryonic stem (ES) cells [2], [3]. It has been shown that the epigenetic status, such as the presence of DNA methylation and histone modifications, changes dramatically during iPS cell generation [4]–[6]. For instance, the promoter regions of *HoxA10* and *Gja8* were reported to be methylated during the reprogramming process [7]. However, *de novo* DNA methyltransferases, Dnmt3a and 3b, are dispensable for the reprogramming of somatic cells to a pluripotent state [8]. On the other hand, the DNA methylation level of the *Oct4* and *Nanog* promoters dramatically decreases during iPS cell generation [1]. Partially reprogrammed iPS cells showed hypermethylation in these regions, suggesting that DNA demethylation is important for the generation of fully reprogrammed cells [6]. However, the mechanism(s) underlying the changes in methylation status are still unclear.

There are considered to be two main possibilities for the mechanism responsible for the DNA demethylation during iPS cell generation. One is ‘passive DNA demethylation’ by the inhibition of the maintenance DNA methyltransferase, Dnmt1, during DNA replication [9]. The other possibility is ‘active DNA demethylation’ mediated by DNA demethylase or a demethylation complex, which was reported to be composed of DNA deaminase and DNA glycosylase [9], [10].

Activation-induced cytidine deaminase (Aid, also known as Aicda) converts methylated cytosine to thymine and unmethylated cytosine to uracil by removing their amine residues [11]. Aid is expressed in B cells upon antigen stimulation and generates point mutations at their Ig locus, which is essential for the initiation of class switch recombination and somatic hypermutation [12], [13]. Recently, several reports suggested that Aid is involved in the DNA demethylation that occurs during the developmental processes in zebrafish and mice [10], [14], while *Aid* ^−/−^ mice develop normally [12]. The DNA methylation level of the *Oct4* and *Nanog* promoters in human fibroblasts were decreased during the reprogramming process after fusion with mouse ES cells. Interestingly, transient suppression of Aid expression has been shown to inhibit this demethylation [15]. Aid is also involved in the DNA demethylation that occurs in the adult mouse brain via the 5-hydroxymethylcytosine generated by Tet1 [16].

Based on these results, we hypothesized that Aid may play an important role in DNA demethylation during iPS cell generation. In this study, we employed a loss of function approach and examined the effects of Aid depletion on the DNA methylation status in mouse iPS cells. Aid depletion did not affect the efficiency of iPS cell generation from the fibroblasts or primary B cells. The characterization of *Aid* ^−/−^ iPS cells showed that they were able to self-renew and had pluripotency. A comprehensive DNA methylation analysis showed few differences between *Aid* ^+/+^ and *Aid* ^−/−^ iPS cells. These results suggest that Aid does not have a crucial function in the DNA demethylation that occurs during the generation of iPS cells.

## Results

### Generation of iPS Cells from *Aid* ^−/−^ Mice

**Figure 1:**
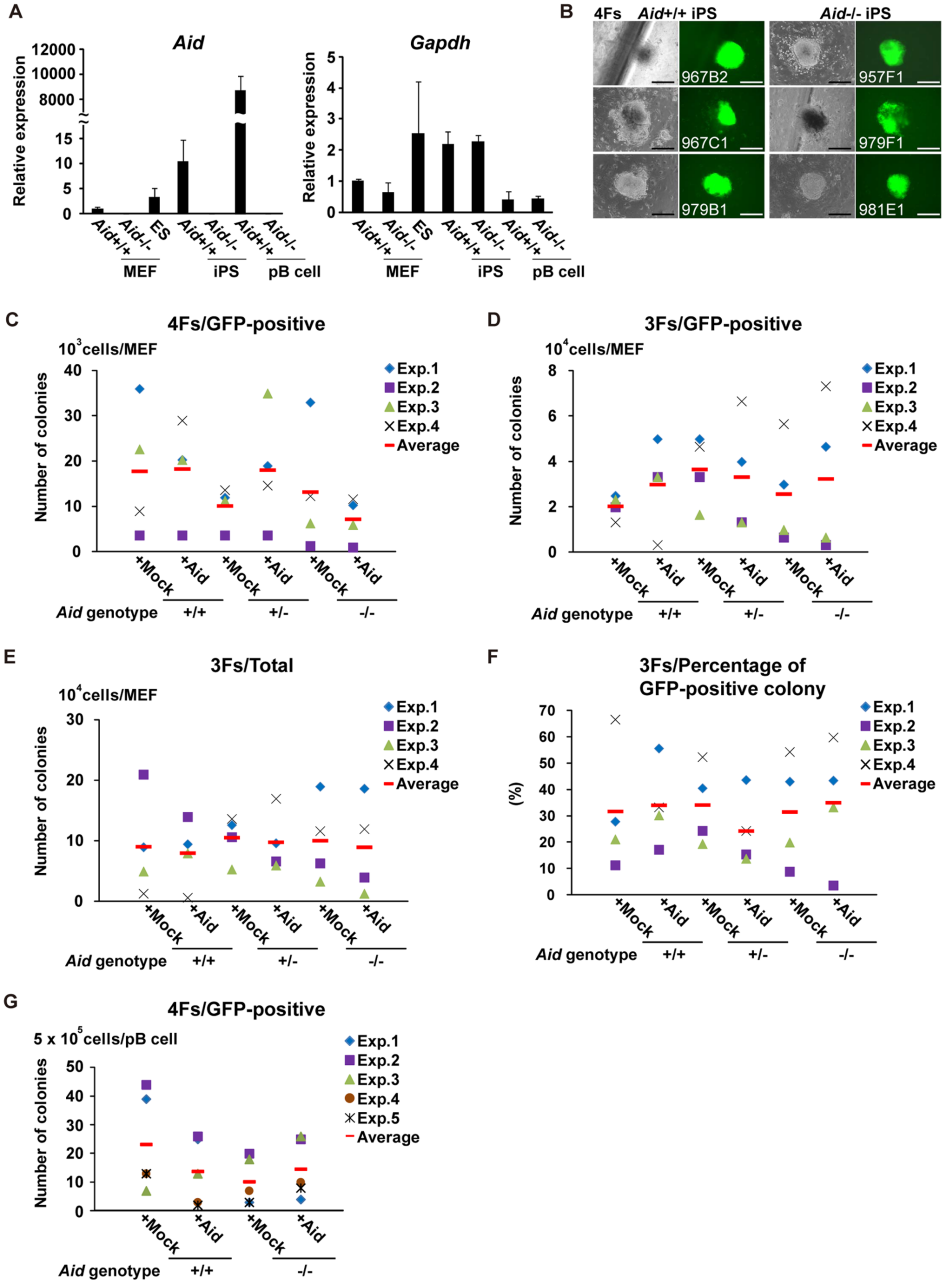
Generation of iPS cells from *Aid* ^−/−^ mice. (A) The relative expression of *Aid* and *Gapdh*. Total RNA was isolated from three ES cell clones (RF8, B6ES and MG1.19), three *Aid* ^+/+^ iPS cell clones (967B2, 967C1 and 979B1), three *Aid* ^−/−^ iPS cell clones (957F1, 979F1 and 979E1), three parental *Aid* ^+/+^ and *Aid* ^−/−^ MEF clones and primary B cells (pB cells), and was used for the quantitative RT-PCR analysis. The data are shown as the average ± SD. (B) The morphology of *Aid* ^+/+^ and *Aid* ^−/−^ iPS colonies 25 days after the introduction of 4 Fs into MEFs. Phase contrast (left column) and GFP fluorescence (right column) images are shown. Scale bars; 200 μm. (C, D) The number of GFP-positive colonies from *Aid* ^+/+^, *Aid* ^+/−^ and *Aid* ^−/−^ MEFs induced by 4 Fs (C) and 3 Fs (D). For each genotype, three different lots of MEFs were used in each experiment, and the experiments were repeated four times. Colonies were counted 25 (4 Fs) and 30 (3 Fs) days after the induction. (E) The number of total colonies from *Aid* ^+/+^, *Aid* ^+/−^ and *Aid* ^−/−^ MEFs subjected to transduction of the 3 Fs with or without Aid. (F) The proportion of GFP-positive colonies out of the total colonies from *Aid* ^+/+^, *Aid* ^+/−^ and *Aid* ^−/−^ MEFs induced by 3 Fs with or without Aid. (G) The number of GFP-positive colonies from *Aid* ^+/+^ and *Aid* ^−/−^ primary B cells induced with 4 Fs. Experiments were repeated five times.

We initially examined the expression of *Aid* in mouse embryonic fibroblasts (MEFs), ES cells and iPS cells by quantitative RT-PCR. The signal for *Aid* was detected in *Aid* ^+/+^ MEFs, ES cells and *Aid* ^+/+^ iPS cells, although their levels were about 9900-, 3000- and 950-fold lower than that of activated primary B cells, respectively (Fig. 1A).

To examine the function of Aid in iPS cell generation, *Aid* ^−/−^ mice expressing a Nanog-GFP reporter were generated by crossing *Aid* ^−/−^ mice [12] and Nanog-GFP mice [2], because Nanog is a marker for iPS cell generation. Then, MEFs were isolated from the mice, and iPS cells were induced by the introduction of Oct3/4, Sox2 and Klf4 with or without c-Myc (4 Fs or 3 Fs, respectively) using retrovirus vectors (Fig. S1A). GFP-positive colonies emerged from the *Aid* ^−/−^ MEFs under both conditions (Fig. 1B).

The resulting colonies were tightly packed and round-shaped, and the cells had a high nuclear/cytoplasm ratio, which was indistinguishable from that of the *Aid* ^+/+^ iPS cells. The number of GFP-positive colonies derived from *Aid* ^−/−^ MEFs was 13.3±13.9 from 10^3^ input cells with 4 Fs and 2.6±2.3 from 10^4^ input cells with 3 Fs, which was comparable to that of *Aid* ^+/+^ (17.8±14.5 and 2.0±0.5) and *Aid* ^+/−^ cells (10.2±4.4 and 3.7±1.5) (Figs. 1C and D). Moreover, the overexpression of Aid did not affect the induction efficiency, even though the expression level was approximately 5,000 higher than that of *Aid* ^+/+^ MEFs (Figs. 1C and D and S2A). The number of total colonies (GFP-positive and negative colonies) and the proportion of GFP-positive colonies in *Aid* ^−/−^ MEFs transduced with 3 Fs were comparable to those of *Aid* ^+/+^ MEFs (Figs. 1E and F).

Although no significant differences were observed in the number of colonies, it is possible that there were differences in the process of iPS cell generation. SSEA1 is a marker for the progression of iPS cell generation [17], [18]. We therefore examined the number of SSEA1-positive cells at several time points after the infection of the 4 Fs (Fig. S3). However, there were no significant differences in the number of SSEA1-positive cells between the *Aid* ^+/+^ and *Aid* ^−/−^ MEFs at any time point.

If Aid has only a limited effect on reprogramming, the high expression of exogenous reprogramming factors might overwhelm the lack of Aid. Therefore, we induced iPS cells with diluted amounts (1 to 1/64) of retrovirus (Fig. S4). The number of GFP-positive colonies generated from Aid^−/−^ MEFs was 14.4±12.0 (1), 9.4±5.8 (1/4), 4.8±3.6 (1/16) and 0±0 (1/64) and that of Aid^+/+^ iPS cells was 8±2.1 (1), 9.4±3.0 (1/4), 4.6±3.4 (1/16) and 0±0 (1/64), showing no statistically significant difference between groups.

The expression level of Aid in MEFs was very low, so it was considered to be plausible that the deletion of Aid would not affect the efficiency of iPS cell generation. Thus, we introduced 4 Fs into primary B cells, because their Aid expression was much higher than that of MEFs. There were no significant differences between *Aid* ^+/+^ (23.2±17.0) and *Aid* ^−/−^ primary B cells (10.2±8.2) in terms of the efficiency of iPS cell generation (Fig. 1G). The overexpression of Aid also did not affect the reprogramming efficiency (Figs. 1G and S2B). Consequently, neither deletion nor overexpression of Aid affected the efficiency of iPS cell generation.

Although the depletion of Aid did not affect the induction efficiency of iPS cells, it is possible that other genes might compensate for the deletion. A recent paper showed that transient knockdown of Aid decreased the efficiency of iPS cell generation [19]. Hence, we tested several shRNA sequences using *Aid* ^+/+^ MEFs, and found four-fold suppression of the expression by shAid#3 (Fig. S5A). However, the addition of shAid#3 did not affect the reprogramming efficiency (Figs. S5B and C). Apobec family genes also have cytidine deamination activity, and were reported to work as a component of the DNA demethylation complex in zebrafish embryos [10]. To address their potential compensatory effects, we introduced 4 Fs into *Aid* ^−/−^ MEFs, together with a dominant negative form of Apobec1, which lacks deaminase activity [20]. Although the expression of the dominant negative form was confirmed by quantitative RT-PCR (Fig. S6A), it did not influence the efficiency of iPS cell generation (Fig. S6B). In addition, the overexpression of Apobec1 itself also did not affect the efficiency (Fig. S6B). These results suggest that Apobec1 did not compensate for the deletion of Aid.

### Characterization of *Aid* ^−/−^ iPS Cells

To examine whether Aid deletion affected the quality of iPS cells, we examined the morphology, proliferation, self-renewal capacity, RNA expression and differentiation potential of *Aid* ^−/−^ iPS cells in detail. *Aid* ^+/+^ and *Aid* ^−/−^ iPS colonies were generated from *Aid* ^+/+^ and *Aid* ^−/−^ MEFs, and were selected from three independent experiments. The clones were passaged four times on feeder cells and two times on gelatin-coated dishes to exclude any contamination of the feeder cells. Subsequently, the RNA and genomic DNA were isolated. The proportions of iPS cell clones showing undifferentiated characteristics throughout the culture period were 90.7±16.2% in *Aid* ^+/+^ iPS cells and 91.7±14.4% in *Aid* ^−/−^ iPS cells (Table S1). Clone independence was confirmed by detecting the genomic integration pattern of retrovirally-introduced Klf4 (Fig. S7). Mature iPS cell clones were selected by the quantification of transgene suppression (Fig. S8), since it is a marker of full reprogramming [2], [6]. There was no statistically significant differences between the *Aid* ^+/+^ and *Aid* ^−/−^ iPS cell clones (Fig. S8). The lack of Aid was confirmed by genomic PCR (Fig. S9). Finally, we selected nine *Aid* ^+/+^ and *Aid* ^−/−^ iPS cell clones for further characterization (Fig. S8).

**Figure 2:**
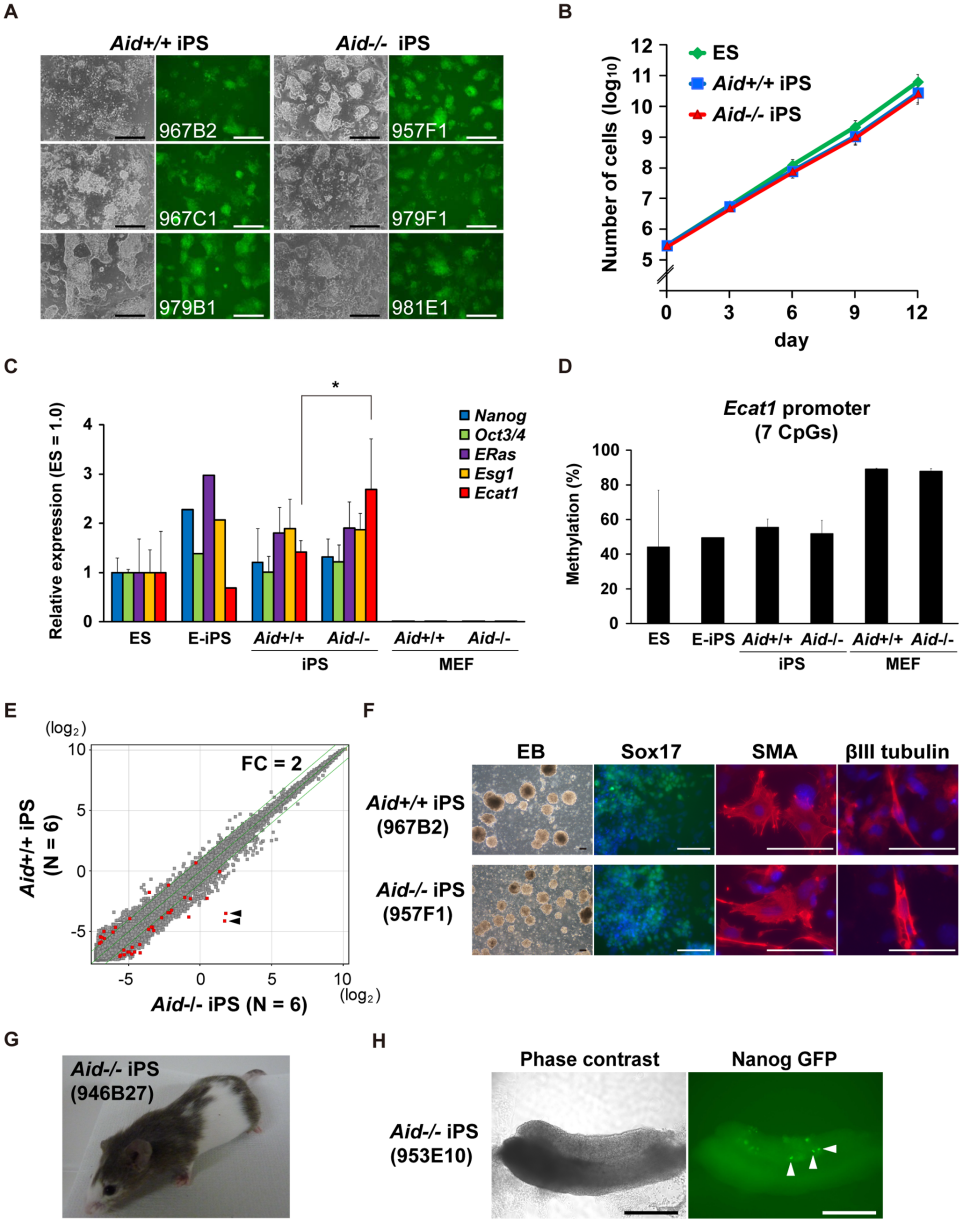
Characterization of the *Aid* ^−/−^ iPS cells. (A) The morphology of the *Aid* ^+/+^ and *Aid* ^−/−^ iPS cells cultured on gelatin-coated dishes (passage 6). Phase contrast (left column) and GFP fluorescence (right column) images are shown. Scale bars; 200 μm. (B) The proliferation of the cells. Three clones of ES cells, *Aid* ^+/+^ iPS cells and *Aid* ^−/−^ iPS cells were passaged every three days (3×10^5^ cells per well of a 6-well plate) on feeder-coated dishes. (C) The relative expression of pluripotent stem cell marker genes. Total RNA was isolated from three ES cell clones, two E-iPS cell clones (20D17 and 178B5), nine *Aid* ^+/+^ iPS cell clones, nine *Aid* ^−/−^ iPS cell clones and three clones each of parental *Aid* ^+/+^ and *Aid* ^−/−^ MEFs. The data were normalized to the level of *Gapdh*. The average of the ES cell clones was set at a relative level of 1 (*, corrected *p*-value <0.05). (D) The DNA methylation level of the *Ecat1* promoter detected by pyrosequencing. (E) Scatter plots showing a comparison of the global gene expression between *Aid* ^+/+^ and *Aid* ^−/−^ iPS cells. The red dots indicate differentially expressed probes (corrected *p*-value <0.05). Green lines indicate two-fold changes. The two most significant probes (arrowheads) were located at the 3′ UTR of Aid and detected immature Aid mRNA in *Aid* ^−/−^ iPS cells, which should be driven by the inserted promoter in the drug resistance cassette. (F) The differentiation of *Aid* ^−/−^ iPS cells *in vitro*. Differentiated *Aid* ^−/−^ iPS cells were stained with antibodies for Sox17, SMA and βIII tubulin. Bars; 100 μm. (G) A chimeric mouse established with *Aid* ^−/−^ iPS cells. Black hair indicates the contribution of the iPS cells. (H) Gonads isolated from 13.5 d.p.c chimeric embryos. GFP-positive cells (arrowheads) indicate the differentiation of *Aid* ^−/−^ iPS cells into PGCs. Bars; 200 μm.

The *Aid* ^−/−^ iPS cells were morphologically indistinguishable from the *Aid* ^+/+^ iPS cells (Fig. 2A). All of the *Aid* ^−/−^ iPS cell clones showed fluorescence of Nanog-GFP. Their doubling time was 16.3±0.7 h, which was comparable to that of *Aid* ^+/+^ iPS cells (16.4±0.7 h, Fig. 2B). When single *Aid* ^−/−^ iPS cells were plated into 96-well plates, 11.3±3.1 wells became positive for Nanog-GFP colonies, similar to the number of *Aid* ^+/+^ iPS cells (12±10.4) (Fig. S10A). The majority of *Aid* ^−/−^ iPS cells in the colonies expressed Nanog-GFP and the Oct3/4 protein (Fig. S10B). These results indicated that *Aid* ^−/−^ iPS cells had the capacity for self-renewal, similar to that observed in *Aid* ^+/+^ iPS cells.

Next, the expression levels of markers of pluripotent stem cells, *Nanog*, endogenous *Oct3/4*, *ERas*, *Esg1* and *Ecat1*, were quantified by RT-PCR. *Aid* ^−/−^ iPS cells exhibited 1.9-fold higher expression of *Ecat1* than in *Aid* ^+/+^ iPS cells, while there were no statistically significant differences in the other genes (Fig. 2C).

We then assessed the DNA methylation status of the *Ecat1* promoter region. The proportion of methylated CpG was 89.0±0.7% in *Aid* ^+/+^ MEFs (Fig. 2D). On the other hand, that of *Aid* ^+/+^ iPS cells and established, well-characterized iPS cells (E-iPS cells), was 55.4±5.0% and 49.4%, respectively, which were similar to that of ES cells (44.0±33.0%) [2], [21]. These results indicated that the promoter region was demethylated during iPS cell generation. The DNA methylation level in *Aid* ^−/−^ iPS cells was 51.7±7.9%, which was comparable to that of the *Aid* ^+/+^ iPS cells, suggesting that the difference in *Ecat1* expression was not due to a change in the DNA methylation level in the *Ecat1* promoter region (Fig. 2D).

Subsequently, we compared the global gene expression profiles of six *Aid* ^+/+^ and six *Aid* ^−/−^ iPS cell clones, and detected 12 downregulated and 26 upregulated probes among a total of 54,497 probes examined in *Aid* ^−/−^ iPS clones (fold change >2, corrected *p*-value <0.05) (Fig. 2E and Table S2). A hierarchal cluster analysis did not show any clear segregation of *Aid* ^+/+^ and *Aid* ^−/−^ iPS cells (Fig. S11). These results demonstrated that there were few differences between *Aid* ^+/+^ and *Aid* ^−/−^ iPS cells in terms of the global gene expression patterns.

To evaluate the differentiation potential of *Aid* ^−/−^ iPS cells, we first performed an *in vitro* differentiation assay. *Aid* ^+/+^ and *Aid* ^−/−^ iPS cells were differentiated through the formation of embryoid bodies (EBs). Subsequently, the expression of marker genes for the three germ layers was examined by immunostaining. Differentiated *Aid* ^−/−^ iPS cells expressed Sox17 (endoderm), smooth muscle actin (SMA) (mesoderm) and βIII tubulin (ectoderm) (Figs. 2F and S12). These results suggested that *Aid* ^−/−^ iPS cells had the capacity to differentiate into all three germ layers *in vitro*. We also performed a teratoma formation assay and confirmed the potential of the cells to differentiate into all three germ layers (data not shown).

We then injected *Aid* ^−/−^ iPS cells into early mouse embryos and tested their contribution to adult chimeras (Fig. 2G). Chimeras could be generated from seven out of eight clones of *Aid* ^−/−^ iPS cells (87.5%) (Table S3). The gonads were isolated from 13.5 days post-coitum embryos to evaluate their differentiation into germline cells. Nanog is a marker of primordial germ cells (PGC) [2], [22], and the existence of GFP-positive cells in the gonads indicated their contribution to the germline (Fig. 2H). As a result, it was concluded that *Aid* ^−/−^ iPS cells have a capacity for self-renewal and pluripotency that is similar to that of *Aid* ^+/+^ iPS cells.

### DNA Methylation Status of *Aid* ^−/−^ iPS Cells

**Figure 3:**
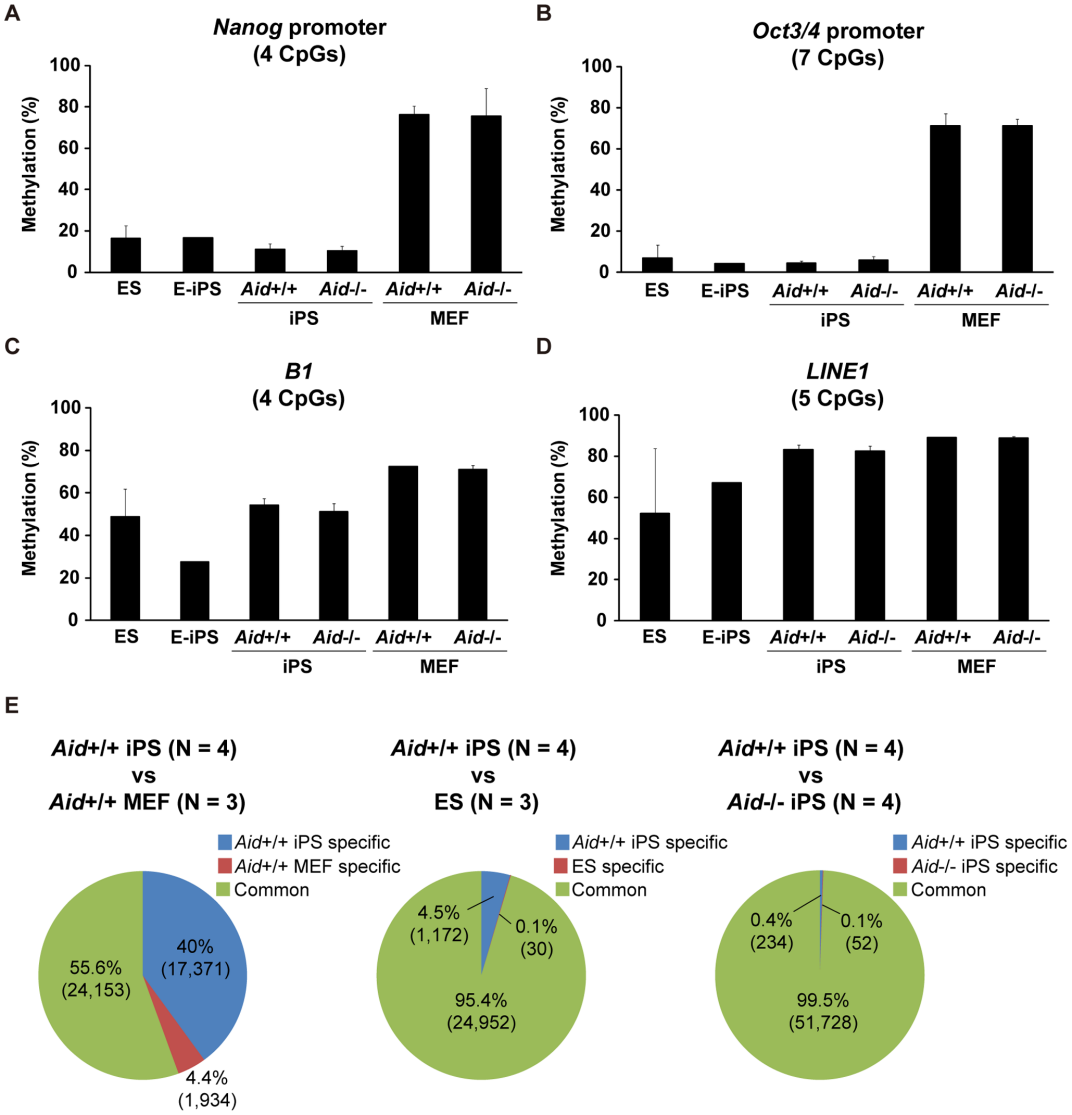
The DNA methylation status of *Aid* ^−/−^ iPS cells. (A–D) The DNA methylation status of the *Nanog* promoter (A), *Oct3/4* promoter (B), B1 (C) and LINE1 (D) detected by pyrosequencing. The iPS cell clones analyzed were the same as those examined in Fig. 2C. The data are represented as the averages ± SD of the clones. (E) The results of the comprehensive DNA methylation analysis with MBD-sequencing. Pie charts show the comparison of the detected methylated regions between *Aid* ^+/+^ iPS cells and *Aid* ^+/+^ MEFs (left), *Aid* ^+/+^ iPS cells and ES cells (middle), and *Aid* ^+/+^ iPS cells and *Aid* ^−/−^ iPS cells (right). DMRs; Differentially methylated regions, CMRs; Commonly methylated regions.

Since AID has been reported to play roles in the demethylation of the human *NANOG* and *OCT3/4* promoters in fusion-mediated reprogramming [15], we analyzed the DNA methylation status of mouse orthologous gene promoters in *Aid* ^−/−^ iPS cells (Figs. 3A and B). The methylation level of the *Nanog* promoter was high (76.2±4.2%) in *Aid* ^+/+^ MEFs, whereas it was low (11.1±2.6%) in *Aid* ^+/+^ iPS cells, as observed in previous reports (Fig. 3A) [1]–[3]. *Aid* ^−/−^ iPS cells also showed 10.3±2.2% methylation, which was not significantly different from that in *Aid* ^+/+^ iPS cells. In the same way, the *Oct3/4* promoter showed hypomethylation in both *Aid* ^+/+^ and *Aid* ^−/−^ iPS cells (Fig. 3B).

We next assessed the DNA methylation level of the B1 repeat and LINE1 sequences [23], [24] to determine the effects of Aid depletion on the global DNA methylation status (Figs. 3C and D). The B1 and LINE1 sequences are distributed all over the genome, and cover 2.6% and 19.2% of the mouse genome, respectively [25]. Although there were variations among ES cells, the DNA methylation level of the B1 repeat sequence in *Aid* ^+/+^ MEFs (72.3±0.3%) was higher than that of *Aid* ^+/+^ iPS cells (54.1±3.2%), thus suggesting a decrease in the methylation during iPS cell generation (Fig. 3C). The B1 repeat regions in *Aid* ^−/−^ iPS cells showed a comparable level of DNA methylation (51.1±4.0%) to that in *Aid* ^+/+^ iPS cells. Similar results were obtained for the LINE1 sequence, although the changes were smaller than those of the B1 repeat (Fig. 3D).

To examine the global DNA methylation status of *Aid* ^−/−^ iPS cells in greater detail, we concentrated the methylated genomic fragments by performing immunoprecipitation using the methyl-CpG binding domain protein (MBD) and analyzed the methylation levels by deep sequencing (MBD-seq) [26]. The fragments were mapped to the mouse genome (average, 79.4±7.6%, Table S4) and subsequently, the peaks of the mapped tags were detected by a MACS algorithm [27]. These peaks defined specific methylated regions. Three or four cell lines were used for each cell type. To evaluate the dispersion of the data on MBD-seq, we first compared two cell samples for each cell type, and examined the proportion of the overlap in methylated regions (Fig. S13A). In the case of three *Aid* ^+/+^ MEFs, the proportions were 69% (*Aid* ^+/+^ MEF#1 vs *Aid* ^+/+^ MEF#2), 62% (*Aid* ^+/+^ MEF#1 vs *Aid* ^+/+^ MEF#3) and 53% (*Aid* ^+/+^ MEF#2 vs *Aid* ^+/+^ MEF#3). On the other hand, those of the *Aid* ^+/+^ iPS cells, *Aid* ^−/−^ iPS cells and ES cells were 60 to 78% (n = 4), 63 to 77% (n = 4) and 27 to 46% (n = 3), respectively. ES cells showed a relatively low overlap. This was likely due to the differences in the original mouse strains among these ES cell clones.

To confirm the validity of our studies, we carried out an experiment to evaluate the reliability of the MBD-seq. From the analysis of *Aid* ^+/+^ iPS cells and parental *Aid* ^+/+^ MEFs, a total of 43,458 methylated regions were detected (Figs. 3E and S13B, Table S5). Among them, the numbers of differentially methylated regions (DMRs) in *Aid* ^+/+^ iPS cells and *Aid* ^+/+^ MEFs were 17,371 (40%) and 1,934 (4.4%), respectively. In the case of *Aid* ^+/+^ iPS cells and ES cells, a total of 26,154 regions were detected (Figs. 3E and S13C, Table S5). The numbers of DMRs in the *Aid* ^+/+^ iPS cells and ES cells were 1,172 (4.5%) and 30 (0.1%), respectively. The proportion of DMRs between *Aid* ^+/+^ iPS cells and *Aid* ^+/+^ MEFs (44.4%) was larger than that in *Aid* ^+/+^ iPS cells and ES cells (4.6%), thus suggesting that the analysis reflected the differences in the cell types.

We next compared *Aid* ^+/+^ iPS cells with *Aid* ^−/−^ iPS cells, and found a total of 52,014 regions (Figs. 3E and S13D, Tables S5 and S6). Almost all regions were commonly methylated regions (CMRs) (99.5%), and only 234 (0.4%) and 52 (0.1%) regions were DMRs in *Aid* ^+/+^ and *Aid* ^−/−^ iPS cells, respectively. Consequently, the MBD-seq exhibited few differences in the global DNA methylation status between *Aid* ^+/+^ and *Aid* ^−/−^ iPS cells.

A pyrosequencing analysis revealed significant differences in the DNA methylation levels at the *Nanog* and *Ecat1* promoter regions between *Aid* ^+/+^ iPS cells and *Aid* ^+/+^ MEFs (Figs. 2D and 3A). However, the MBD-seq analysis of *Aid* ^+/+^ MEF-DMRs compared with *Aid* ^+/+^ iPS cells did not include either of these promoters, although there were some mapped reads detected at the Nanog promoter in *Aid* ^+/+^ MEFs (Fig. S13E). One possible explanation for this different result is that MBD-seq is a method based on immunoprecipitation, which can be affected by the density of CpGs [28].

## Discussion

Our findings demonstrated that Nanog-GFP-positive iPS cells could be generated from *Aid* ^−/−^ mice. The deletion, knockdown and overexpression of Aid did not affect the efficiency of iPS cell generation. Based on the results of our characterization, *Aid* ^−/−^ iPS cells did not have any major defects in their capacity for self-renewal or pluripotency. In addition, there were few significant differences between *Aid* ^+/+^ and *Aid* ^−/−^ iPS cells in the comprehensive DNA methylation assay. These results suggest that Aid does not have a crucial function in DNA demethylation during iPS cell generation.

Previous studies have indicated that Aid is involved in DNA demethylation in the early embryos of zebrafish, in mouse PGCs and in the reprogramming of human fibroblasts fused with mouse ES cells [10], [14], [15]. These studies led us to hypothesize that Aid was also involved in the DNA demethylation that occurs during iPS cell generation. However, our results did not support this hypothesis. One possible reason was thought to be compensation by other genes.

Bhutani et al. reported that transient knockdown of Aid in the initial phase of iPS cell generation decreased the efficiency of mouse iPS cell generation, while genomic deletion of Aid did not affect it [19]. Based on these results, they suggested that there may be compensation mechanisms for genomic depletion. We also examined the effects of transient knockdown by the shRNA sequences employed in their report. However, this transient knockdown did not affect the efficiency of iPS cell generation in our hands. Habib et al. reported similar results [29], thereby supporting our present findings.

The effect of Aid overexpression is also controversial. Bhutani et al. showed that the overexpression of human AID enhanced the generation of mouse iPS cells [19]. On the contrary, the addition of mouse Aid did not affect the reprogramming efficiency in our current study. Kumar et al. and Habib et al. also examined the effects of mouse Aid overexpression on the proportion of Oct3/4-positive cells during iPS cell generation, and did not observe any significant effect [29], [30]. Further supporting our results, even in mature B cells expressing abundant Aid (Fig. 1A), Aid was not involved in determining the DNA methylation status [31], [32]. The homology of human and mouse Aid is 92% at the amino acid sequence level. Although the homology is relatively high, it is still possible that the differences might influence the effects of Aid overexpression on the reprogramming.

After the formation of iPS colonies, the efficiency of establishing stable cell lines from *Aid* ^−/−^ MEFs (91.7±14.4%) was comparable to that from *Aid* ^+/+^ MEFs (90.7±16.2%, Table S1) in the present study. On the other hand, Kumar et al. reported that five out of 12 *Aid* ^−/−^ iPS cell colonies (41.7%), but zero out of 13 *Aid* ^+/+^ iPS cell colonies differentiated during the culture period [30], which suggested the involvement of Aid in the maintenance of pluripotency. This instability, however, could not be rescued by the ectopic expression of Aid. They discussed that there may be unknown molecular mechanism(s), beyond that mediated by Aid, that contribute to the maintenance of pluripotency. It should be noted that *Aid* ^−/−^ ES cells can be established and give rise to chimeric mice [12]. In addition, constitutive expression of Aid did not disrupt general mouse development [33]. These reports indicated that Aid may not have a crucial function in the maintenance of pluripotency in ES cells.

There were several controversial studies about the function of Aid in factor-mediated reprogramming, while all of these studies showed the generation of iPS cell colonies from *Aid* ^−/−^ mice. One possible explanation for the diverse results is the differences in the experimental settings among studies, such as the construction of the reprogramming vectors, culture medium, the method used to evaluate the reprogramming efficiency and the mouse strain. The deaminase activity of Aid is reported to be regulated by its phosphorylation at Thr27 and Ser38 [34], [35], as well as its cellular localization [36], [37]. The experimental settings can also affect the activity of Aid.

The expression of *Ecat1* in *Aid* ^−/−^ iPS cells was 1.9-fold higher than that of *Aid* ^+/+^ iPS cells (Fig. 2C). The function of Ecat1 in pluripotent stem cells has not been well studied. Therefore, it is difficult to speculate on the biological meaning of the difference in expression. The methylation level of the promoter seemed to have little effect on this distinct expression. The involvement of Aid in the change in Ecat1 expression needs to be studied.

For the comprehensive DNA methylation assay, we employed MBD-seq in combination with the detection of methylated regions by using a MACS algorithm. It should be noted that this detection is affected by the types of cells being examined. The number of methylated regions in *Aid* ^+/+^ iPS cells (94,615±13,994) (Table S4) was two times higher than that in *Aid* ^+/+^ MEFs (47,278±11,215). These results were different from those in a previous report showing that there were no significant differences between the levels of global DNA methylation in ES cells and fibroblasts examined by bisulfite sequencing [38]. We found a lot of small peaks in the *Aid* ^+/+^ MEFs compared with the *Aid* ^+/+^ iPS cells in our study (Fig. S13F). One possible cause of the small peaks is populational heterogeneity. MEFs consist of variety of cell types, while iPS cells are clonal uniform cells. It is possible that the small peaks were recognized as background differences by the MACS algorithm, and influenced its sensitivity of detection. On the other hand, in the case of comparisons between the same cell type, such as *Aid* ^+/+^ iPS cells and *Aid* ^−/−^ iPS cells, the number of detected regions was 94,615±13,994 and 85,968±10,880, which was not significantly different.

Our current study suggested that there is likely some yet to be elucidated mechanism(s) responsible for demethylation during iPS cell induction other than that involving Aid. The DNA methylation status is known to be related to the histone modifications [39]. Recently, histone modifiers like Suv39H1, the NuRD complex and Utx were reported to function during iPS cell generation [40]–[42]. Therefore, if there are unknown DNA demethylation enzyme(s), they may work with the interactions of such histone modifiers. One possible candidate would be Tet2, an enzyme converts 5-methylcytosine to 5-hydroxymethycytosine, which is recently reported to function in DNA demethylation during iPS cell generation [43]. It is also important to examine the involvement of passive DNA demethylation, which dilutes methylated DNA in a cell division-dependent manner. Treatment of the reprogramming cells with inhibitors of DNA methylation and histone modification, such as 5-azacytidine and trichostatin A, would help to investigate these factors in greater detail.

As described above, Aid initiates class switch recombination and somatic hypermutation by generating point mutations at Ig locus in B cells [12], [13]. In addition, Aid is also reported to generate point mutations and/or double strand breaks at non-Ig locus, for example BCL6, MYC, PIM1 and PAX5, which are related to the cancer genesis [44]–[46]. These results suggest that Aid potentially generate genomic mutation during iPS cell generation. Several reports have indicated that iPS cell clones have genomic mutations that occurred during the reprograming process and/or during cell culture [47], [48]. On the contrary, one paper showed that most of the genomic mutations in iPS cells were preexisting in the parental somatic cells [49]. Although it is still unclear whether iPS cells develop genomic mutations during the reprogramming process, the effect of Aid on mutations should be carefully examined.

In summary, we herein showed that mouse iPS cells can be normally established in the absence of a functional Aid gene. The *Aid* ^−/−^ iPS cells were similar to the *Aid* ^+/+^ iPS cells in terms of their capacity for self-renewal and pluripotency, as well as their DNA methylation status. These results suggest that Aid does not have any crucial function in DNA demethylation during iPS cell generation.

## Materials and Methods

### Plasmid Constructs

The coding regions of mouse Aid (NM_009645.2, NCBI) and mouse Apobec1 (NM_001134391, NCBI) were cloned from mouse ES cells by RT-PCR. The PCR products were sequenced and subcloned into pENTR-D-TOPO (Invitrogen) and recombined with pMXs-gw [1] using LR recombinase according to manufacturer’s instructions (Invitrogen). Mouse dominant-negative Apobec1 (H61K/C93S/C96S) [20] was generated by PCR-based site-directed mutagenesis. To generate lentivirus vectors encoding doxycycline-inducible reprogramming factors, TRE, the Gateway cassette (Invitrogen) and rtTA2s-M2 (Clontech) were introduced into a pLKO.1 backbone (#10878, Addgene). Then coding sequences of Oct3/4, Sox2, Klf4 and c-Myc were inserted by the LR reaction to make pLV-TRE-rtTA2s-M2-Oct3/4, -Sox2, -Klf4 and -c-Myc. psPAX2 (#12260) and pMDG.2 (#12259) were obtained from Addgene. The primers used for the construction of plasmids are listed in Table S7.

### Mice

All mice used in this study were bred and sacrificed appropriately following the code of ethics of the Animal Research Committee of Kyoto University. The animal care and experimental procedures used in this study were approved by the Animal Research Committee of Kyoto University and were carried out according to the Regulation on Animal Experimentation at Kyoto University (approval ID: 2–10). To generate *Aid* ^−/−^ Nanog-GFP reporter mice, *Aid* ^−/−^ mice (C57BL6) [12] were crossed with Nanog-GFP mice [2].

### Establishment of MEFs

The establishment of MEFs was performed from individual 13.5 d.p.c embryos, as described previously, with some modifications [50]. Briefly, heads and gastrointestinal tract tissues were removed from the embryos. The embryos were then dissected using a pair of scissors and dissociated using trypsin. The cell suspensions were plated onto a 100-mm gelatin-coated dish. Three days after the plating, the MEFs were expanded to three 100-mm dishes. Three days after the passage, the MEFs were trypsinized, divided into six vials and frozen as a stock. *Aid* ^+/+^ and *Aid* ^−/−^ MEFs derived from male mice, which were homozygous for the Nanog-GFP reporter, were used for iPS cell generation. The littermates of *Aid* ^+/+^ and *Aid* ^−/−^ mice were generated by crossing Aid heterozygous KO male and female mice, and MEFs were also generated from these mice.

### Cell Culture

RF8 ES cells (129S4 background) [51], B6 ES cells (C57BL/6 background) [52] and MG1.19 (129/Ola background) [53] cells were maintained in ES medium (DMEM containing 15% FBS, 2 mM L-glutamine, 1×nonessential amino acids, 1.1 mM 2-mercaptoetahanol, 50 units/mL penicillin and 50 μg/mL streptomycin) with LIF on feeder cells, as described previously [2]. As a source for LIF, we used the conditioned medium from Plat-E cells [54] that had been transduced with a LIF-expressing vector. The *Aid* ^+/+^ and *Aid* ^−/−^ iPS cells and E-iPS cells (20D17 and 178B5) [2], [21] were maintained in ES medium with LIF and 1.5 μg/mL puromycin on feeder cells. MEFs were maintained in fibroblast medium (DMEM containing 10% FCS, 50 units/mL penicillin and 50 μg/mL streptomycin) as described previously [50]. The Plat-E cells were maintained in fibroblast medium with 1 μg/mL puromycin and 10 μg/mL blasticidin. Primary B cells were maintained in RPMI 1640 supplemented with 10% FCS, 10 mM 2-mercaptoethanol, 5% NTCT, 20 mM HEPES, 2 mM L-glutamine, 100 units/mL penicillin and 100 μg/mL streptomycin, as described previously, with some modifications [55].

### Generation and Establishment of Mouse iPS Cells from MEFs Using Retroviruses

The generation of mouse iPS cells from MEFs was performed using retroviruses as described previously, with some modifications [50]. Briefly, Plat-E cells were seeded at 3.6×10^6^ cells per 100 mm dish. On the following day, reprogramming factors were independently introduced into Plat-E cells using the FuGENE 6 (Roche) transfection reagent. After 24 h, the medium was replaced with fibroblast medium. MEFs were seeded in six-well plates at 2×10^5^ cells per well. The following day, virus-containing supernatants from the Plat-E cultures were recovered and filtered using a 0.45 μm pore size cellulose acetate filter. Equal volumes of virus-containing supernatants were mixed together (for example, viruses for Oct3/4, Sox2, Klf4, c-Myc and Aid), then the MEFs were incubated in virus supernatant containing polybrene at a final concentration of 4 μg/mL for 24 h. Four days after transduction, the MEFs were reseeded onto dishes covered with feeder cells. As the combinations of reprogramming factors affect the efficiency of iPS cell generation [21], [56], we re-seeded 10^3^ cells per 100 mm dish for 4 Fs and 10^4^ cells for 3 Fs. One day after the re-seeding, the medium was changed to ES cell medium with LIF. The puromycin selection (1.5 μg/mL) was started from day 14 for 4 Fs and from day 21 for 3 Fs. The number of iPS colonies was counted on day 25 for 4 Fs and on day 30 for 3 Fs.

To establish mouse iPS cells, GFP-positive iPS colonies were mechanically picked up and passaged on feeder cells [50]. To examine the number of SSEA1-positive cells, we reseeded 1.5×10^3^ MEFs onto gelatin-coated 6-well plates four days after the transduction of 4 Fs and cultured them in ES medium. Eight, 12, 16, 20, 24 and 28 days after the transduction, we counted the number of total cells and analyzed the proportion of SSEA1-positive cells by flow cytometry The number of SSEA1-positive cells was calculated by multiplying the number of total cells by the proportion of SSEA1-positive cells. The antibody used for flow cytometry was an Alexa Fluor 647-conjugated anti-SSEA1 antibody (sc-21702 AF647, Santa Cruz Biotechnology).

### Generation of Mouse iPS Cells from MEFs with Lentiviruses

The 293T cells were seeded at 3.6×10^6^ cells per 100-mm dish. On the following day, reprogramming factors were independently introduced into 293T cells with psPAX2 and pMDG.2 using the FuGENE 6 transfection reagent. After 24 h, the medium was replaced with fibroblast medium containing 10 μM forskolin. Two days after the removal of the transfection reagent, virus-containing supernatants from the 293T cells were recovered and filtered with a 0.45 μm pore size cellulose acetate filter. To enrich the lentiviruses, PEG-*it* Virus Precipitation Solution (System Biosciences) was added, and the mixture was kept at 4°C for 24 h according to the manufacturer’s protocol. Finally, a two-fold enriched lentivirus solution was prepared.

For iPS cell generation, equal volumes of lentiviruses which encoded Oct3/4, Sox2, Klf4 and c-Myc were mixed together. MEFs were seeded in six-well plates at 2×10^5^ cells per well one day before the transduction. The following day, MEFs were incubated in medium containing the viruses and polybrene at a final concentration of 8 μg/mL for 24 h. One day after the transduction, the virus supernatant was removed and changed to ES medium containing doxycycline at a final concentration of 2 μg/mL. Four days after transduction, the MEFs were reseeded onto dishes with feeder cells. The number of iPS colonies was counted on day 30.

### Isolation of Primary B Cells

Primary B cells were isolated from mouse spleens by immunomagnetic depletion with anti-CD43 MicroBeads (Miltenyi Biotech) [45]. The harvested cells were stimulated in the presence of 25 μg/mL LPS (Roche) and 50 ng/mL IL-4 (Sigma-Aldrich) for three days. After the stimulation, RNA was isolated for a further analysis.

### Generation of Mouse iPS Cells from Primary B Cells

CD43-negative primary B cells were isolated from mouse spleens and stimulated in the presence of 25 μg/mL LPS (Roche) and 50 ng/mL IL-4 (Sigma-Aldrich) for 24 h. Then, 10^6^ cells were cultured in 2 mL of the medium containing retroviruses encoding reprogramming factors with LPS and IL-4 in one well of a six-well plate for two days. After the infection, the medium was changed to ES medium, and 5×10^5^ cells were reseeded onto SNL feeder cells in the 100 mm dishes. On days five and seven, 10 mL of ES medium was added to the dish, and the medium was replaced on day nine. Twenty-five days after the B cell isolation, the number of GFP-positive colonies was counted.

### Gene Knockdown

The hairpin sequences for Aid and GFP were cloned into the pLKO.1 vector. The preparation of lentiviruses was performed as described above. One day before the transduction, MEFs were seeded in 60-mm dishes at a concentration of 4×10^5^ cells/dish. On the following day, MEFs were incubated in medium containing the virus (es) and polybrene (8 μg/mL) for 24 h. Two days after the infection, puromycin was added to the medium for the selection of shRNA-expressing cells. Three days after the addition of puromycin, the total RNA was isolated. The oligo DNAs used for the hairpin sequences are listed in Table S7.

### Southern Blot Analysis

Genomic DNA (6 μg) purified from cultured cells was digested with *Bam*HI and *Eco*RI, separated on 0.8% agarose gels and transferred to nylon membranes (Amersham). The membranes were incubated with digoxigenin (DIG)-labeled DNA probes in DIG Easy Hyb Buffer (Roche) at 42°C with constant agitation. After washing, an alkaline phosphatase-conjugated anti-DIG antibody (1∶10,000, Roche) was added to the membrane. Signals were raised by CDP-star (Roche) and detected by the LAS3000 imaging system (FUJIFILM). The Klf4 cDNA probe was generated using the DIG DNA labeling mix (Roche). The primers used in this experiment are listed in Table S7.

### RNA Isolation and Reverse Transcription

To exclude any contamination of the feeder cells, ES cells and iPS cells were passaged twice on gelatin-coated dishes. Subsequently, the total RNA was isolated from the cells using the TRIzol reagent (Invitrogen) according to the manufacturer’s instructions. The extracted RNA was treated with TURBO DNase (Ambion) for 30 minutes at 37°C to eliminate the genomic DNA contamination. RNA (1 μg) was reverse transcribed with RevaTra Ace (TOYOBO) using oligo-dT primers in a 20 μL reaction volume. The cDNA was diluted with 80 μL distilled water, and 2 μL of the dilution was used for PCR assays.

### Quantitative PCR

Quantitative PCR was performed with the StepOnePlus Real-Time PCR system (Applied Bio Systems) and SYBR *premix Ex Taq*II (Takara) according to the manufacturer’s instructions. The primers used for the PCR reaction are listed in Table S7.

### Western Blot Analysis

The Western blot analyses were performed as described previously, with some modifications [57]. Briefly, 5×10^5^ primary B cells were lysed with 50 μL 1 × NuPAGE LDS Sample Buffer (Life Technologies) according to manufacturer’s protocol. Subsequently, we used 7 μL of the sample for electrophoresis on 12% SDS-polyacrylamide gel, and transferred the proteins to a polyvinylidine difluoride membrane (Millipore). The blot was blocked with TBST (20 mM Tris-HCl, pH 7.6, 136 mM NaCl, and 0.1% Tween-20) containing 5% skim milk. The membranes were incubated in Can Get Signal Immunoreaction Enhancer Solution I (TOYOBO) with primary antibody solution at 4°C overnight. Then, the membrane was incubated in Can Get Signal Immunoreaction Enhancer Solution II (TOYOBO) with a horseradish peroxidase (HRP)-conjugated secondary antibody for 1 hr at room temperature. Signals were detected with the ECL Prime Western Blotting Detection System (GE Healthcare) and the LAS3000 imaging system (FUJIFILM, Japan). The antibodies used for the Western blotting analysis were anti-Aid (1∶1000, mAID-2, eBioscience), anti-β actin (1∶1000, AC15, Sigma-Aldrich), anti-mouse IgG-HRP (1∶1000, #7076, Cell Signaling) and anti-rat IgG-HRP (1∶1000, #712-035-153, Jackson ImmunoResearch).

### Clonogenic Assay

Nanog-GFP-positive and DAPI-staining-negative single iPS cells were plated into each well of 96-well plates after being sorted by a FACS Aria instrument (BD). Seven days after plating, the cells were fixed and stained with an anti-Oct3/4 antibody (1∶100, C-10, Santa Cruz Biotechnology) and DAPI (Sigma-Aldrich). The secondary antibody used was Alexa Fluor 647-conjugated goat anti-mouse IgG (1∶500, Life Technologies). Subsequently, the number of Nanog-GFP positive colonies was counted.

### *In vitro* Differentiation

For the EB formation, iPS cells were harvested by trypsinization and transferred to bacterial culture dishes in ES medium without LIF. After seven days, the EBs were photographed and plated into gelatin-coated dishes for another three days. The cells were fixed with PBS containing 4% paraformaldehyde for 15 minutes at room temperature. Immunostaining was performed as described previously [57]. The primary antibodies used were an anti-Sox17 antibody (1∶200, R&D Systems), anti-SMA antibody (1∶500, 1A4, DAKO) and anti-βIII tubulin (1∶1000, TUJ1, Covance). The secondary antibodies used were Alexa Fluor 488-conjugated donkey anti-Goat IgG (1∶500, Life Technologies) for Sox17, and Alexa Fluor 546-conjugated donkey anti-mouse IgG (1∶500, Life Technologies) for SMA and βIII tubulin.

### Chimera Formation

Ten to fifteen iPS cells were injected into MCH (ICR)-derived blastocysts. After the injection, the blastocysts were transplanted into the uteri of pseudo-pregnant mice.

### DNA Microarray

Total RNA was labeled with Cy3 and hybridized to the Whole Mouse Genome Microarray (Agilent, catalog no. 28005) as described previously, with some modifications [2]. The data were analyzed with the GeneSpring GX version 11 software program (Agilent). Quantile normalization (75%) was performed. The microarray data are available from the Gene Expression Omnibus (GEO), and appear under the accession number GSE51955.

### Pyrosequencing

Bisulfite treatment was performed using the EZ DNA methylation-Gold Kit (ZYMO RESEARCH) with 2 μg of input genomic DNA. The treated DNA was eluted with 20 μL elution buffer, and the concentration was adjusted to 5 ng/μL with distilled water. Subsequently, we performed PCR using 1 μL of the bisulfite-converted genome. PCR products were sequenced using the PyroMark Q96 ID (Qiagen) according to the manufacturer’s protocol. To assess the DNA methylation status of the B1 repeat and LINE1 sequences, the ADS010/Mouse B1 Element Methylation Analysis (EpigenDX) and ADS685/Mouse Line-1 Global Methylation Assay (EpigenDX) were used. The primers used in this experiment are listed in Table S7.

### MBD-sequencing

Genomic DNA was sonicated to 100–300 bp fragments with a Covaris E210 (Covaris) device. Methylated DNA was enriched with an EpiXplore Methylated DNA Enrichment Kit (Takara) according to the manufacturer’s protocol. The enriched methylated DNA was sequenced with a Hiseq2000 device (Illumina). The data were mapped to the mouse genome (UCSC assembly mm9, NCBI built 37) using the BWA 0.5.9rc1 software program (http://bioinformatics.oxfordjournals.org/content/25/14/1754). Peaks were detected by the MACS version 1.4.1 software program (http://genomebiology.com/2008/9/9/r137), with a default *p*-value threshold of *p*<10^−5^ [58]. For the comparison of two cell types, we extracted the overlapping methylated regions among one cell type and examined whether the overlapping regions were also detected in each of the samples of the other cell type, one by one. Subsequently, the regions detected in all samples of one cell type but not in any samples of another cell type were defined as differentially methylated regions (DMRs). When the regions were detected in all samples of both cell types, we named them commonly methylated regions (CMRs). The extraction of overlapping methylated regions was done by a modified method based on intersectBed, which is a component of the BEDTools software program [59]. The annotation of the methylated regions was performed according to the RefSeq database. The data are available from the Gene Expression Omnibus (GEO), under the accession number GSE52136.

### Statistical Analyses

The data are shown as the averages and standard deviations of clones or experiments. Student’s *t-*test was used for the statistical analysis. For multiple testing correction for the microarray analysis and RT-PCR analysis of pluripotent stem cell marker genes, the Benjamini & Hochberg correction was used.

## Supporting Information

Figure S1 A schematic diagram showing the schedule of mouse iPS cell induction from MEFs and primary B cells. (A) A schematic diagram of the schedule of mouse iPS cell induction from MEFs. MEFs, mouse embryonic fibroblasts; DMEM, Dulbecco’s modified Eagle medium; FBS, fetal bovine serum; LIF, leukemia inhibitory factor. (B) A schematic diagram of the schedule of mouse iPS cell induction from primary B cells. LPS, lipopolysaccharide; IL-4, Interleukin-4.
(PDF)

Figure S2 Confirmation of Aid overexpression. (A) Total RNA was isolated from MEFs which were induced by 4 Fs, together with Aid. The data were normalized to the level of *Gapdh* and the control was set at a relative level of 1. The data are the averages ± SD of three independent experiments. (B) The results of a Western blot analysis of Aid in *Aid* ^+/+^ and *Aid* ^−/−^ primary B cells induced by 4 Fs together with Aid.
(PDF)

Figure S3 The number of SSEA1-positive cells generated during iPS cell generation. *Aid* ^+/+^ and *Aid* ^−/−^ MEFs were transfected with 4 Fs on day zero and re-seeded onto gelatin-coated 6-well plates on day four (1.5×10^3^ cells). Subsequently, the number of SSEA1-positive cells was examined by flow cytometry on day 8, 12, 16, 20, 24 and 28. The data are the averages ± SD of three independent experiments.
(PDF)

Figure S4 The efficiency of iPS cell generation from *Aid* ^−/−^ MEFs with various expression levels of reprogramming factors. *Aid* ^+/+^ and *Aid* ^−/−^ MEFs were infected with various amounts (1 to 1/64) of retrovirus 4 Fs, and the number of Nanog-GFP-positive colonies was counted 25 days after the infection. The data are the averages ± SD of five independent experiments.
(PDF)

Figure S5 Effects of Aid knockdown on the efficiency of iPS cell generation. (A) The knockdown efficiency of shAids. Lentiviruses encoding shRNA sequences for Aid were infected into MEFs. From days two to five after the infection, puromycin selection was performed for shRNA expression. The total RNA was extracted five days after the infection. The expression of Aid was examined by quantitative RT-PCR. The data were normalized to *Gapdh,* and the level in cells transfected with the scrambled shRNA was set at a relative level of 1. The data are the averages ± SD of three independent experiments. *, *P*<0.05. (B) The schedule of Aid knockdown during iPS cell generation. On day 0, a mixture of lentiviruses containing doxycycline (dox)-inducible Oct3/4, Sox2, Kfl4 and c-Myc, and constitutive shRNA, were transfected into MEFs. On day 1, dox was added to the culture medium to induce the expression of reprogramming factors. The shRNA-expressing cells were selected by puromycin treatment from days 2 to 4. The MEFs were then re-seeded onto SNL feeder cells on day 4. The number of GFP-positive colonies was counted 30 days after the infection. (C) The effects of Aid knockdown on the efficiency of iPS cell generation. 4 Fs were transfected into *Aid* ^+/+^ and *Aid* ^−/−^ MEFs along with shRNAs. The number of GFP-positive colonies was counted 30 days after the infection. The data are shown as the averages ± SD of three independent experiments.
(PDF)

Figure S6 Effects of Apobec1 and its dominant negative form on the efficiency of iPS cell generation. (A) The expression of Apobec1 (Apo1) and the dominant negative form of Apobec1 (Apo1DN) in MEFs was examined by quantitative RT-PCR. The data were normalized to *Gapdh,* and the control (Cont) was set at a relative level of 1. The data are the averages ± SD of the three independent experiments. (B) The number of GFP-positive colonies from *Aid* ^+/+^, *Aid* ^+/−^ and *Aid* ^−/−^ MEFs induced by 4 Fs, and transfected with Apo1 or Apo1DN, 25 days after the induction.
(PDF)

Figure S7 The results of a Southern blot analysis of the transgene integration with a *Klf4* cDNA probe. The arrowhead indicates the endogenous *Klf4* allele. *Aid* ^+/+^ iPS cell clones, 967B2 and 967B3 and 967C2 and 967C3; and *Aid* ^−/−^ iPS cell clones, 979F4 and 979F5, 981E1 and 981E2 and 981E4 and 981E7, were apparently the same clones based on their integration patterns (asterisk).
(PDF)

Figure S8 The relative expression levels of transgenes. Total RNA was isolated from Fbx15 reporter iPS cells (Fbx-iPS) [1], established iPS cells (20D17 and 178B5), *Aid* ^+/+^ iPS cell clones and *Aid* ^−/−^ iPS cell clones, and was used for a quantitative RT-PCR analysis. Each experiment was repeated two times, and the averages are shown. The data were normalized to *Gapdh,* and the data for Fbx-iPS cells was set at a relative level of 1. The third and fourth bars from the left side show the averages of the *Aid* ^+/+^ and *Aid* ^−/−^ iPS clones, respectively. The error bars represent the SD of the clones. The arrowhead indicates the clones selected for characterization.
(PDF)

Figure S9 The results of a genotyping analysis of the *Aid* ^+/+^ and *Aid* ^−/−^ iPS cell clones. Genomic DNA was isolated from *Aid* ^+/+^ iPS cell clones and *Aid* ^−/−^ iPS cell clones. The genotyping analysis was performed by PCR. The primers used for this experiment are listed in Table S7.
(PDF)

Figure S10 Clonogenic assay. (A) The number of Nanog-GFP positive colonies in 96-well plates. Single *Aid* ^+/+^ and *Aid* ^−/−^ iPS cells were plated into 96-well plates, and the number of Nanog-GFP-positive colonies was counted after seven days. The data are the averages ± SD of three iPS cell clones. (B) The expression of the Oct4 and Nanog-GFP proteins. *Aid* ^+/+^ and *Aid* ^−/−^ iPS cell colonies were stained with an antibody for Oct3/4, along with 4', 6-Diamidino-2-Phenylindole (DAPI). Bars; 100 μm.
(PDF)

Figure S11 Hierarchical clustering. The hierarchical clustering analysis of gene expression was performed using all detected probes.
(PDF)

Figure S12 *In vitro* differentiation of *Aid* ^−/−^ iPS cells. *Aid* ^+/+^ (967C1 and 979B1) and *Aid* ^−/−^ iPS cell clones (979F1 and 981E1) were differentiated *in vitro* through the formation of EBs, and were stained with antibodies for Sox17, SMA and βIII tubulin. Bars; 100 μm.
(PDF)

Figure S13 MBD-sequencing. (A) The proportion of overlapping methylated regions between biological replicates. The proportion was calculated by dividing the number of overlapping regions by the number of total regions detected in the two samples. (B–D) Representative methylated regions identified by the comparison of *Aid* ^+/+^ MEFs and *Aid* ^+/+^ iPS cells (B), ES cells and *Aid* ^+/+^ iPS cells (C) and *Aid* ^+/+^ and *Aid* ^−/−^ iPS cells (D). RPM; Reads per million mapped reads. (E) The number of mapped reads at the Nanog and Ecat1 promoter regions in *Aid* ^+/+^ MEFs and *Aid* ^+/+^ iPS cells. Asterisks indicate the regions examined by pryosequencing in Figs. 2D (Nanog promoter) and 3A (Ecat1 promoter). (F) Representative small peaks in *Aid* ^+/+^ MEFs.
(PDF)

Table S1 The efficiency of establishing *Aid* ^−/−^ iPS cells.
(PDF)

Table S2 A list of the differentially expressed probes between *Aid* ^+/+^ and *Aid* ^−/−^ iPS cells.
(PDF)

Table S3 A summary of the blastocyst injections.
(PDF)

Table S4 A summary of the MBD-seq findings.
(PDF)

Table S5 The classification of the methylated regions detected by MBD-seq.
(PDF)

Table S6 A list of the methylated regions detected by MBD-seq (*Aid* ^+/+^ iPS cells vs. *Aid* ^−/−^ iPS cells).
(XLS)

Table S7 The primers used in the present study.
(PDF)
