# Correction: Generation and Characterization of Induced Pluripotent Stem Cells from Aid-Deficient Mice

**DOI:** 10.1371/journal.pone.0119209

**Published:** 2015-03-18

**Authors:** The PLOS ONE Staff

In Fig. 1B, phase contrast and GFP fluorescence images of 967B2, 957F1 and 979F1 are incorrect. Please view the correct Fig. 1 here.

**Fig 1:**
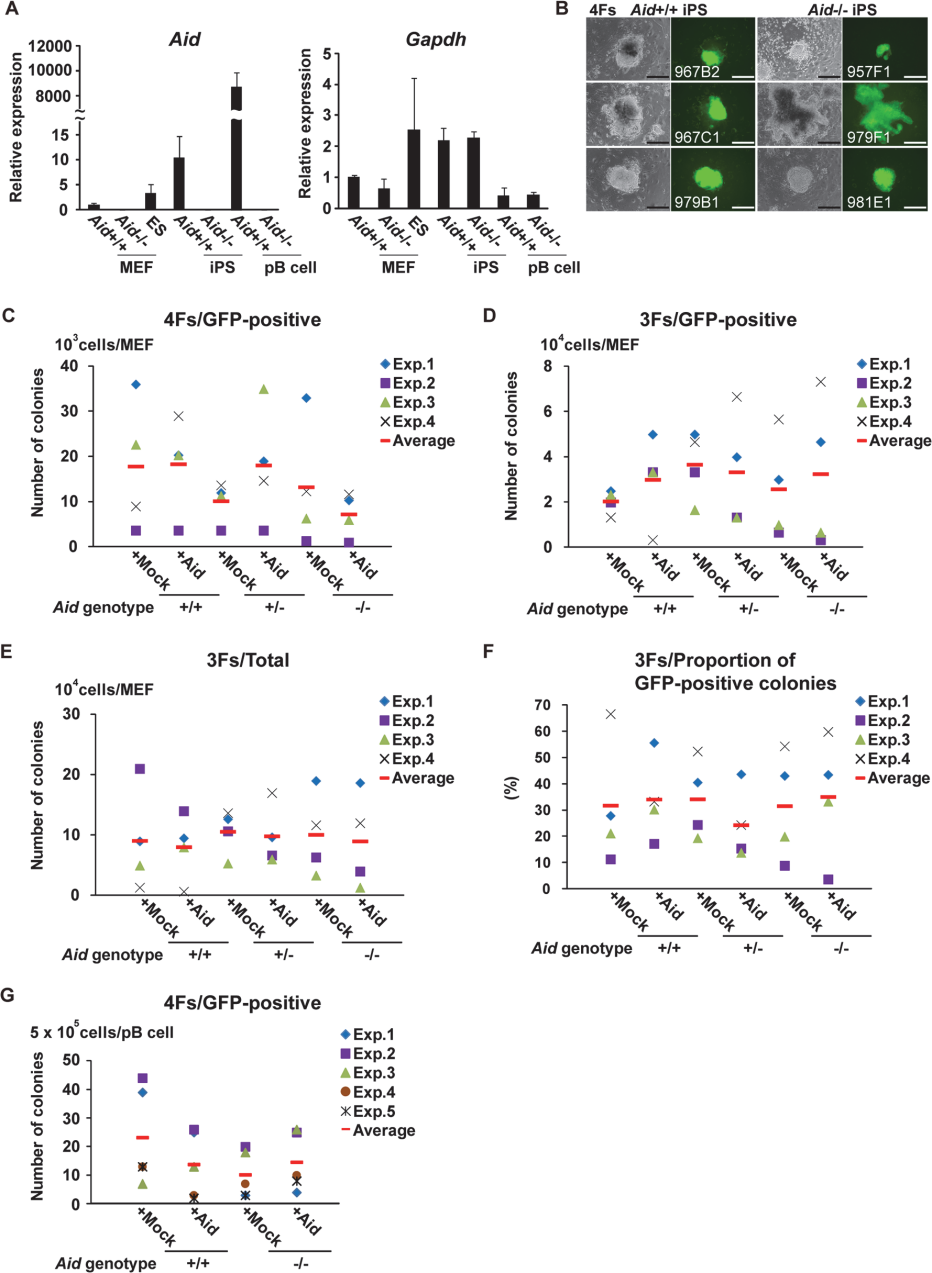
Generation of iPS cells from *Aid* ^−/−^ mice. (A) The relative expression of *Aid* and *Gapdh*. Total RNA was isolated from three ES cell clones (RF8, B6ES and MG1.19), three *Aid* ^+/+^ iPS cell clones (967B2, 967C1 and 979B1), three *Aid* ^−/−^ iPS cell clones (957F1, 979F1 and 979E1), three parental *Aid* ^+/+^ and *Aid* ^−/−^ MEF clones and primary B cells (pB cells), and was used for the quantitative RT-PCR analysis. The data are shown as the average ± SD. (B) The morphology of *Aid* ^+/+^ and *Aid* ^−/−^ iPS colonies 25 days after the introduction of 4Fs into MEFs. Phase contrast (left column) and GFP fluorescence (right column) images are shown. Scale bars; 200 μm. (C, D) The number of GFP-positive colonies from *Aid* ^+/+^, *Aid* ^+/−^ and *Aid* ^−/−^ MEFs induced by 4Fs (C) and 3Fs (D). For each genotype, three different lots of MEFs were used in each experiment, and the experiments were repeated four times. Colonies were counted 25 (4Fs) and 30 (3Fs) days after the induction. (E) The number of total colonies from *Aid* ^+/+^, *Aid* ^+/−^ and *Aid* ^−/−^ MEFs subjected to transduction of the 3Fs with or without Aid. (F) The proportion of GFP-positive colonies out of the total colonies from *Aid* ^+/+^, *Aid* ^+/−^ and *Aid* ^−/−^ MEFs induced by 3Fs with or without Aid. (G) The number of GFP-positive colonies from *Aid* ^+/+^ and *Aid* ^−/−^ primary B cells induced with 4Fs. Experiments were repeated five times.
