# Supplementary figures and images for: Generation and Characterization of Induced Pluripotent Stem Cells from Aid-Deficient Mice
Source: PLoS One. 2014 Apr 9;9(4):e94735. doi: 10.1371/journal.pone.0094735 (PMC3981863; doi:10.1371/journal.pone.0094735)

Figure S1

A

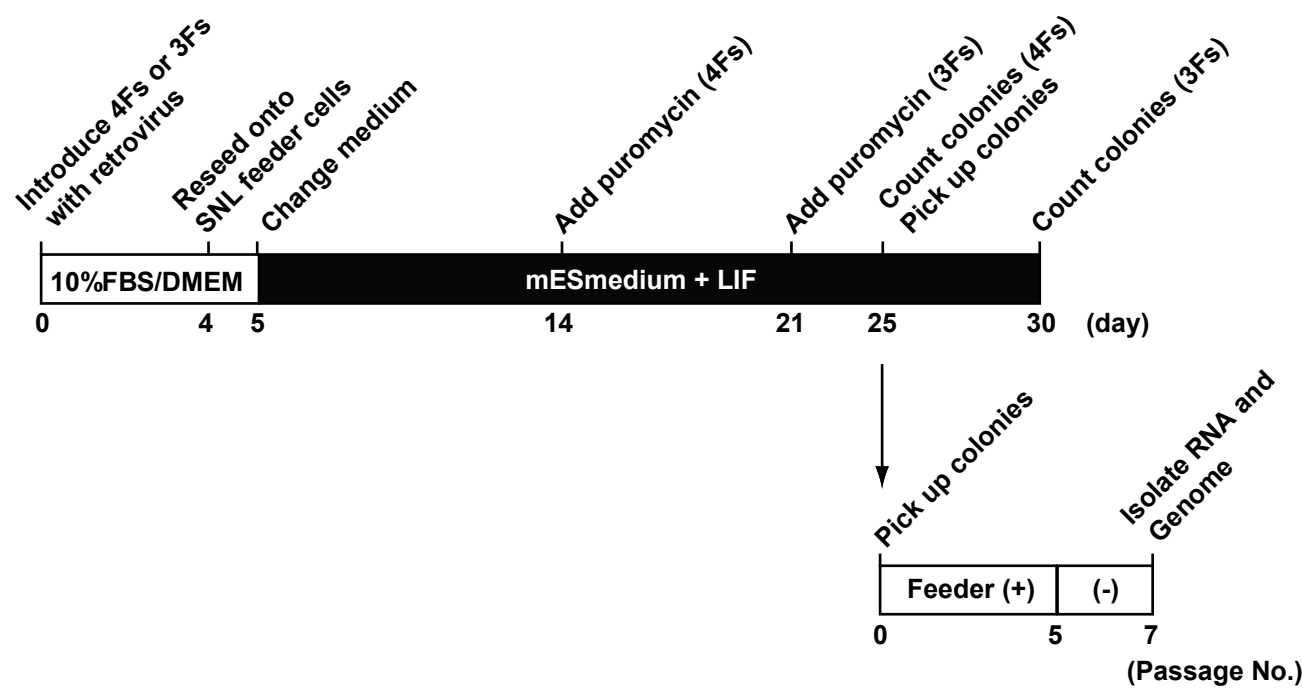

B

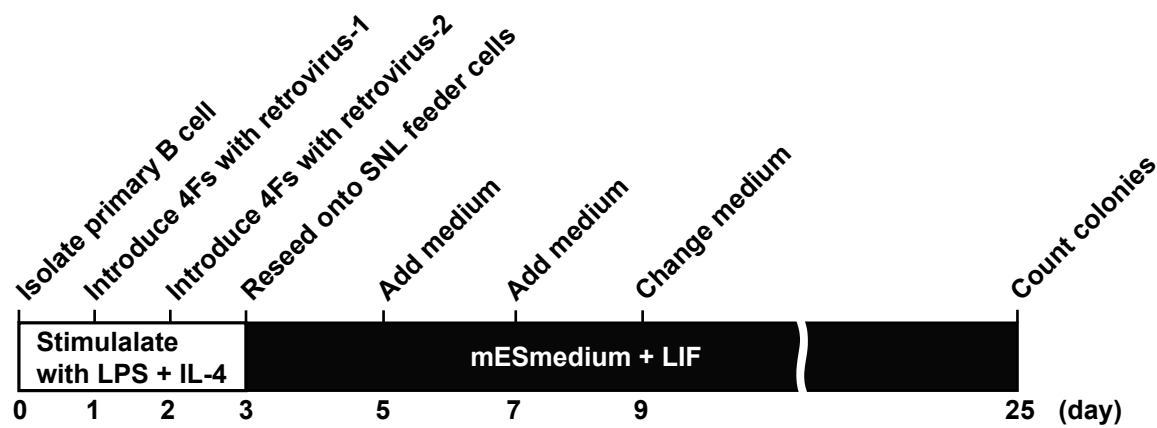

Supplement: Figure S1 — A schematic diagram showing the schedule of mouse iPS cell induction from MEFs and primary B cells. (A) A schematic diagram of the schedule of mouse iPS cell induction from MEFs. MEFs, mouse embryonic fibroblasts; DMEM, Dulbecco’s modified Eagle medium; FBS, fetal bovine serum; LIF, leukemia inhibitory factor. (B) A schematic diagram of the schedule of mouse iPS cell induction from primary B cells. LPS, lipopolysaccharide; IL-4, Interleukin-4. (PDF) [file pone.0094735.s001.pdf]

Figure S2

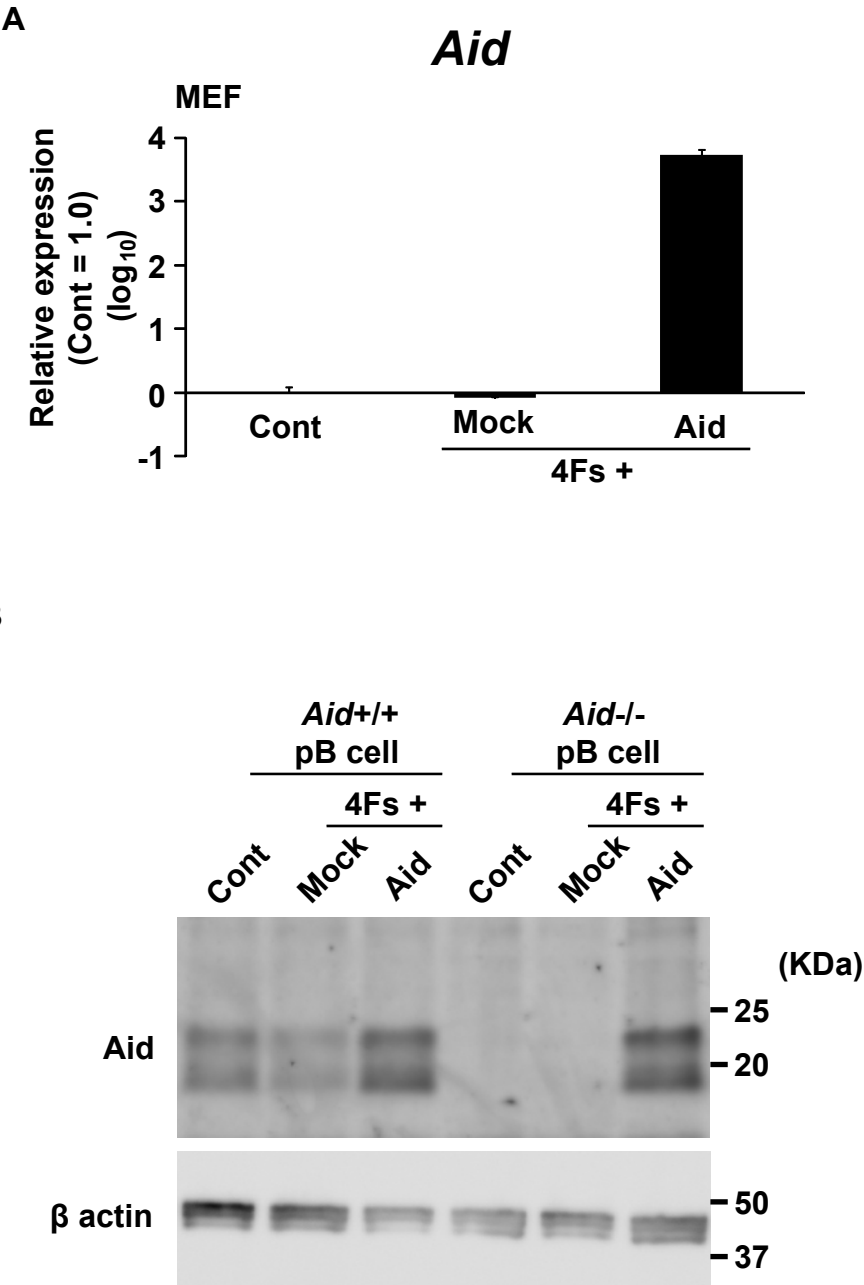

Supplement: Figure S2 — Confirmation of Aid overexpression. (A) Total RNA was isolated from MEFs which were induced by 4 Fs, together with Aid. The data were normalized to the level of Gapdh and the control was set at a relative level of 1. The data are the averages ± SD of three independent experiments. (B) The results of a Western blot analysis of Aid in Aid +/+ and Aid −/− primary B cells induced by 4 Fs together with Aid. (PDF) [file pone.0094735.s002.pdf]

Figure S3

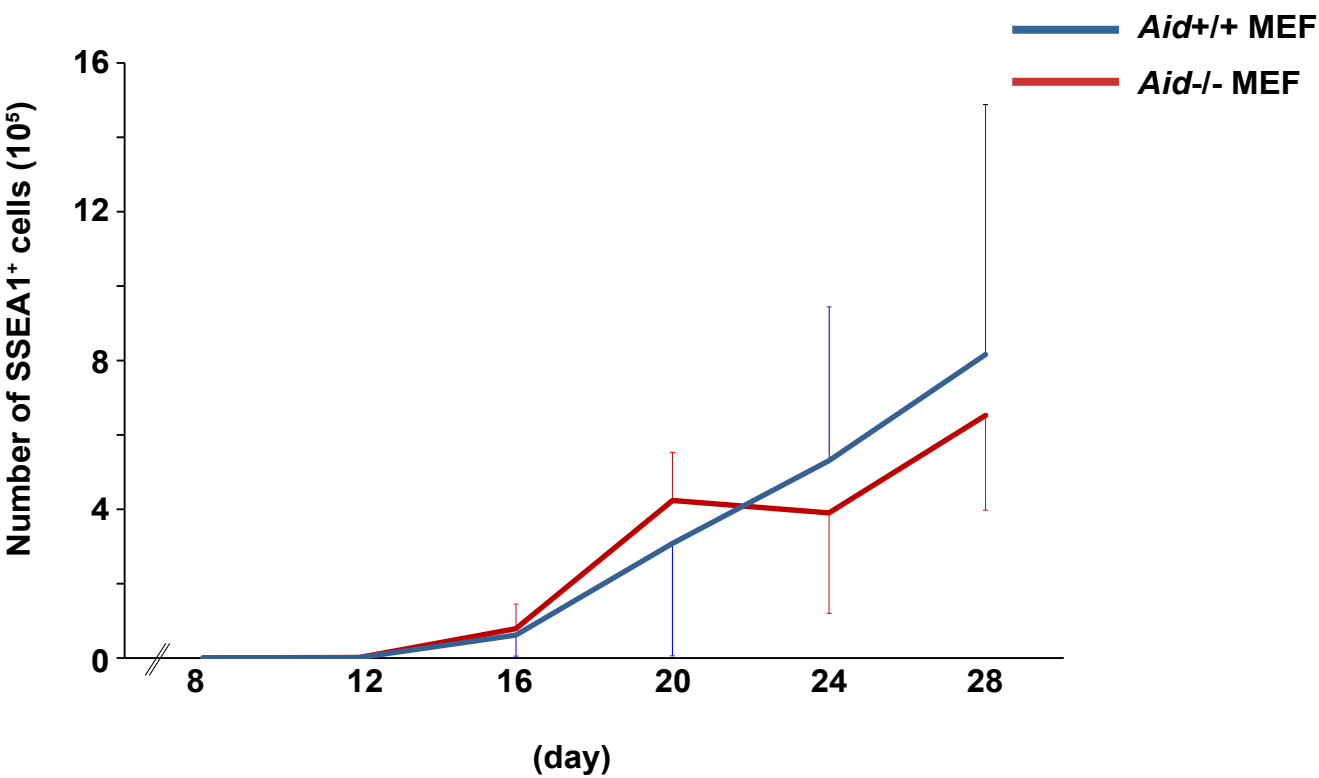

Supplement: Figure S3 — The number of SSEA1-positive cells generated during iPS cell generation. Aid +/+ and Aid −/− MEFs were transfected with 4 Fs on day zero and re-seeded onto gelatin-coated 6-well plates on day four (1.5×10^3^ cells). Subsequently, the number of SSEA1-positive cells was examined by flow cytometry on day 8, 12, 16, 20, 24 and 28. The data are the averages ± SD of three independent experiments. (PDF) [file pone.0094735.s003.pdf]

Figure S4

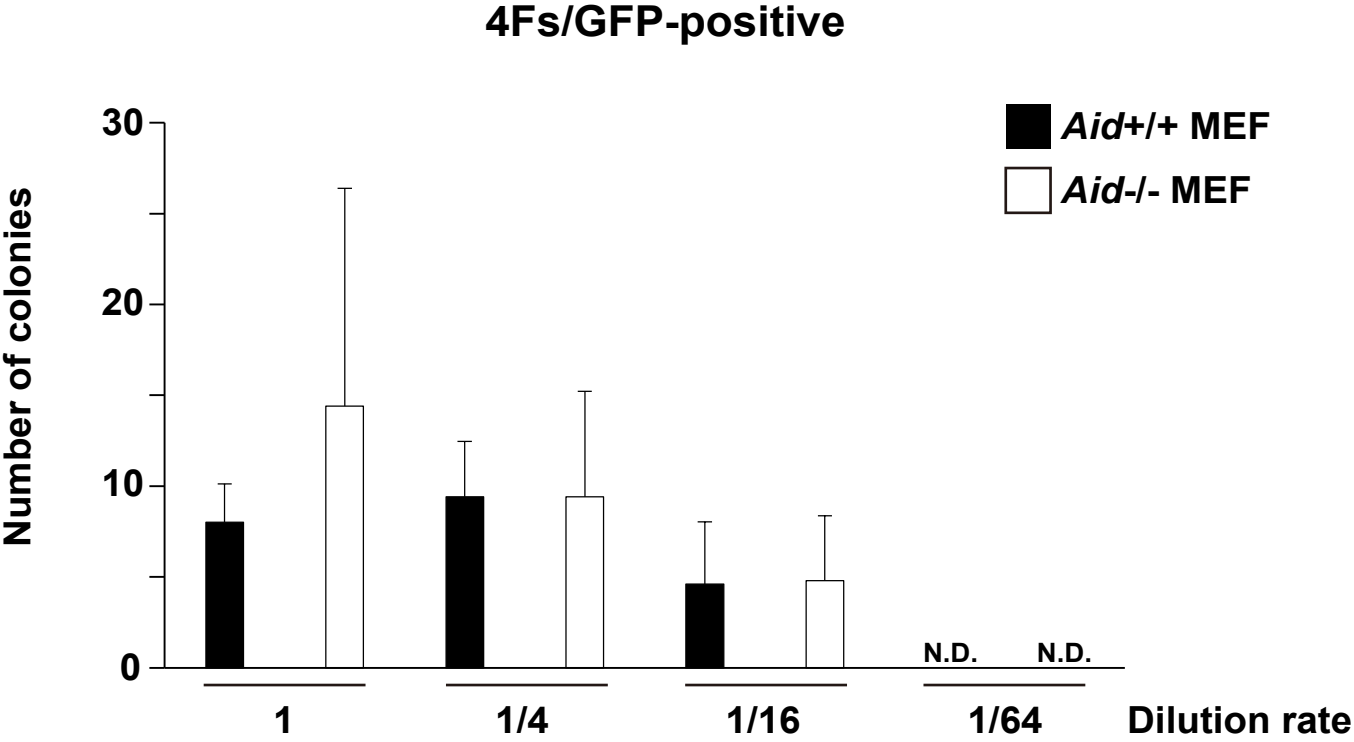

Supplement: Figure S4 — The efficiency of iPS cell generation from Aid −/− MEFs with various expression levels of reprogramming factors. Aid +/+ and Aid −/− MEFs were infected with various amounts (1 to 1/64) of retrovirus 4 Fs, and the number of Nanog-GFP-positive colonies was counted 25 days after the infection. The data are the averages ± SD of five independent experiments. (PDF) [file pone.0094735.s004.pdf]

Figure S5

A

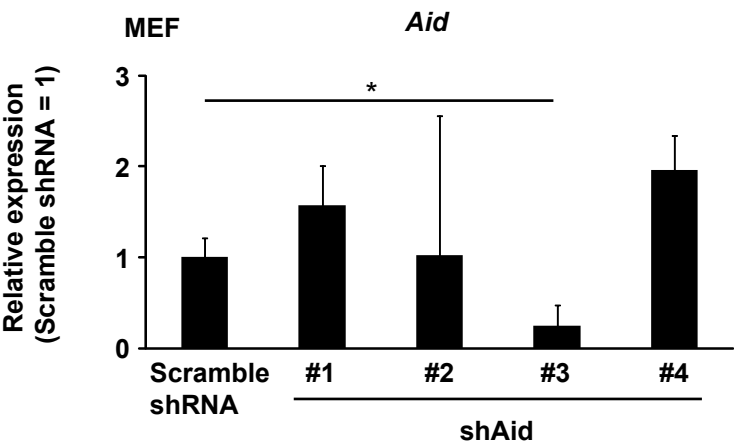

B

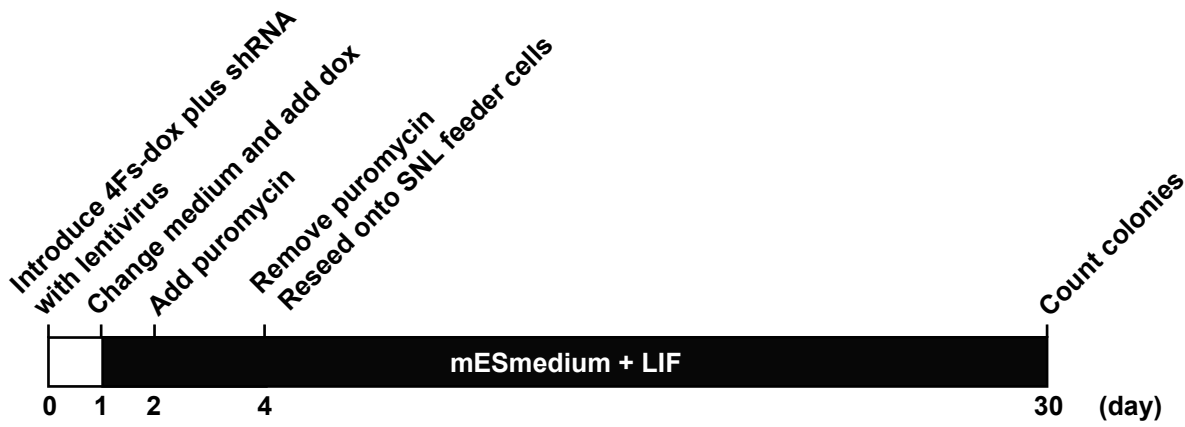

C

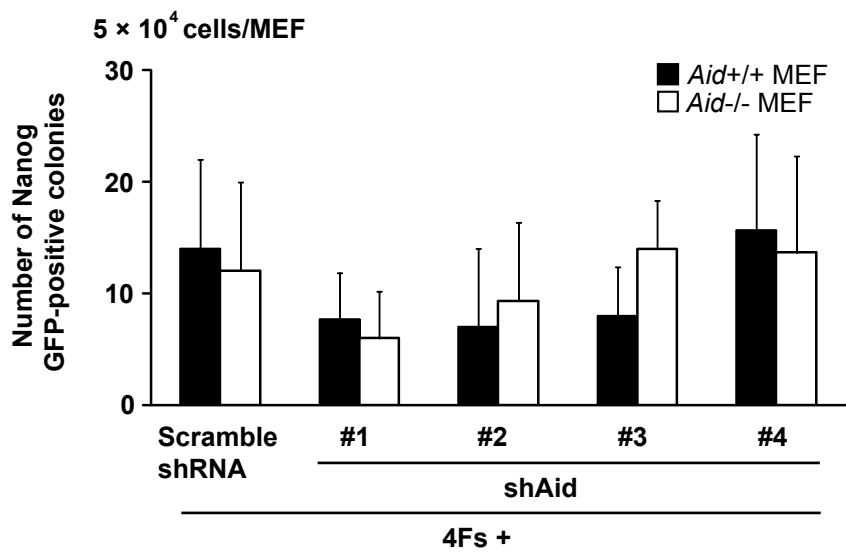

Supplement: Figure S5 — Effects of Aid knockdown on the efficiency of iPS cell generation. (A) The knockdown efficiency of shAids. Lentiviruses encoding shRNA sequences for Aid were infected into MEFs. From days two to five after the infection, puromycin selection was performed for shRNA expression. The total RNA was extracted five days after the infection. The expression of Aid was examined by quantitative RT-PCR. The data were normalized to Gapdh, and the level in cells transfected with the scrambled shRNA was set at a relative level of 1. The data are the averages ± SD of three independent experiments. *, P<0.05. (B) The schedule of Aid knockdown during iPS cell generation. On day 0, a mixture of lentiviruses containing doxycycline (dox)-inducible Oct3/4, Sox2, Kfl4 and c-Myc, and constitutive shRNA, were transfected into MEFs. On day 1, dox was added to the culture medium to induce the expression of reprogramming factors. The shRNA-expressing cells were selected by puromycin treatment from days 2 to 4. The MEFs were then re-seeded onto SNL feeder cells on day 4. The number of GFP-positive colonies was counted 30 days after the infection. (C) The effects of Aid knockdown on the efficiency of iPS cell generation. 4 Fs were transfected into Aid +/+ and Aid −/− MEFs along with shRNAs. The number of GFP-positive colonies was counted 30 days after the infection. The data are shown as the averages ± SD of three independent experiments. (PDF) [file pone.0094735.s005.pdf]

Figure S6

A

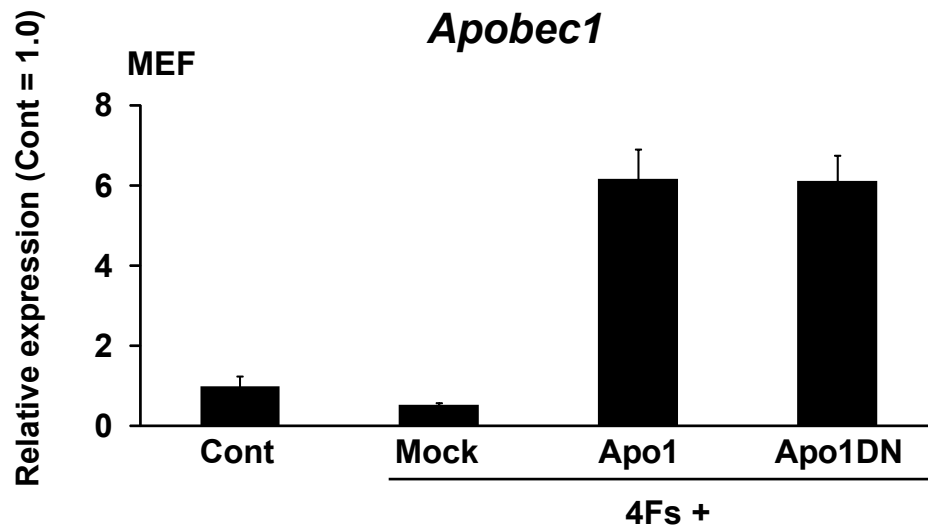

B

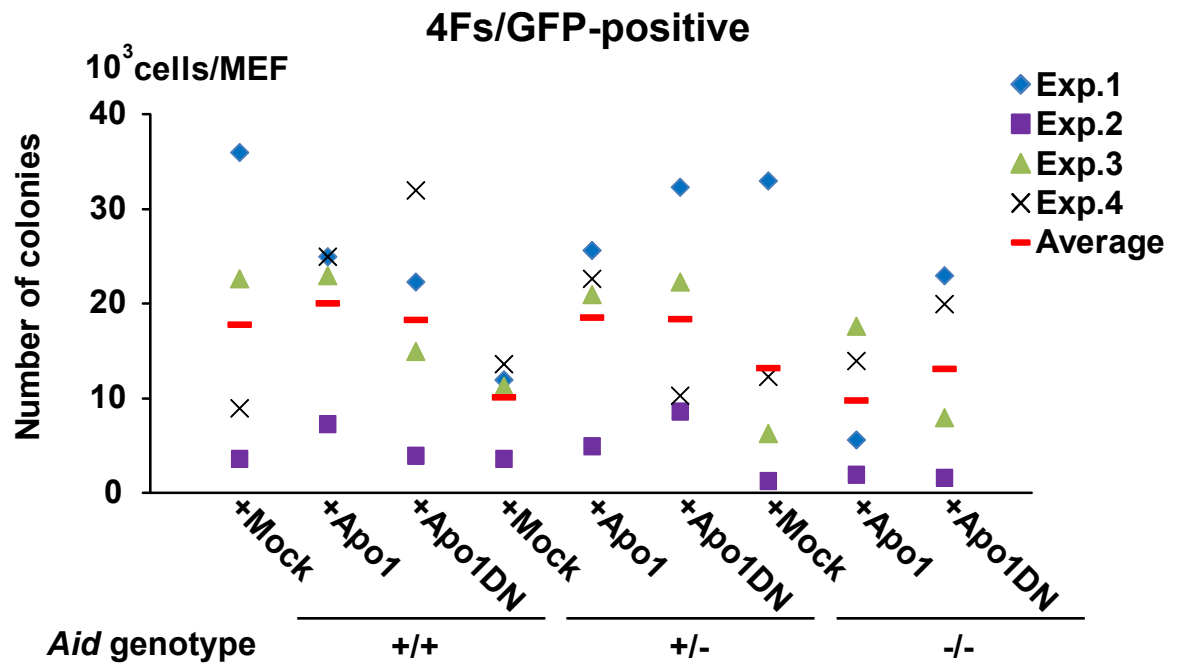

Supplement: Figure S6 — Effects of Apobec1 and its dominant negative form on the efficiency of iPS cell generation. (A) The expression of Apobec1 (Apo1) and the dominant negative form of Apobec1 (Apo1DN) in MEFs was examined by quantitative RT-PCR. The data were normalized to Gapdh, and the control (Cont) was set at a relative level of 1. The data are the averages ± SD of the three independent experiments. (B) The number of GFP-positive colonies from Aid +/+, Aid +/− and Aid −/− MEFs induced by 4 Fs, and transfected with Apo1 or Apo1DN, 25 days after the induction. (PDF) [file pone.0094735.s006.pdf]

Figure S7

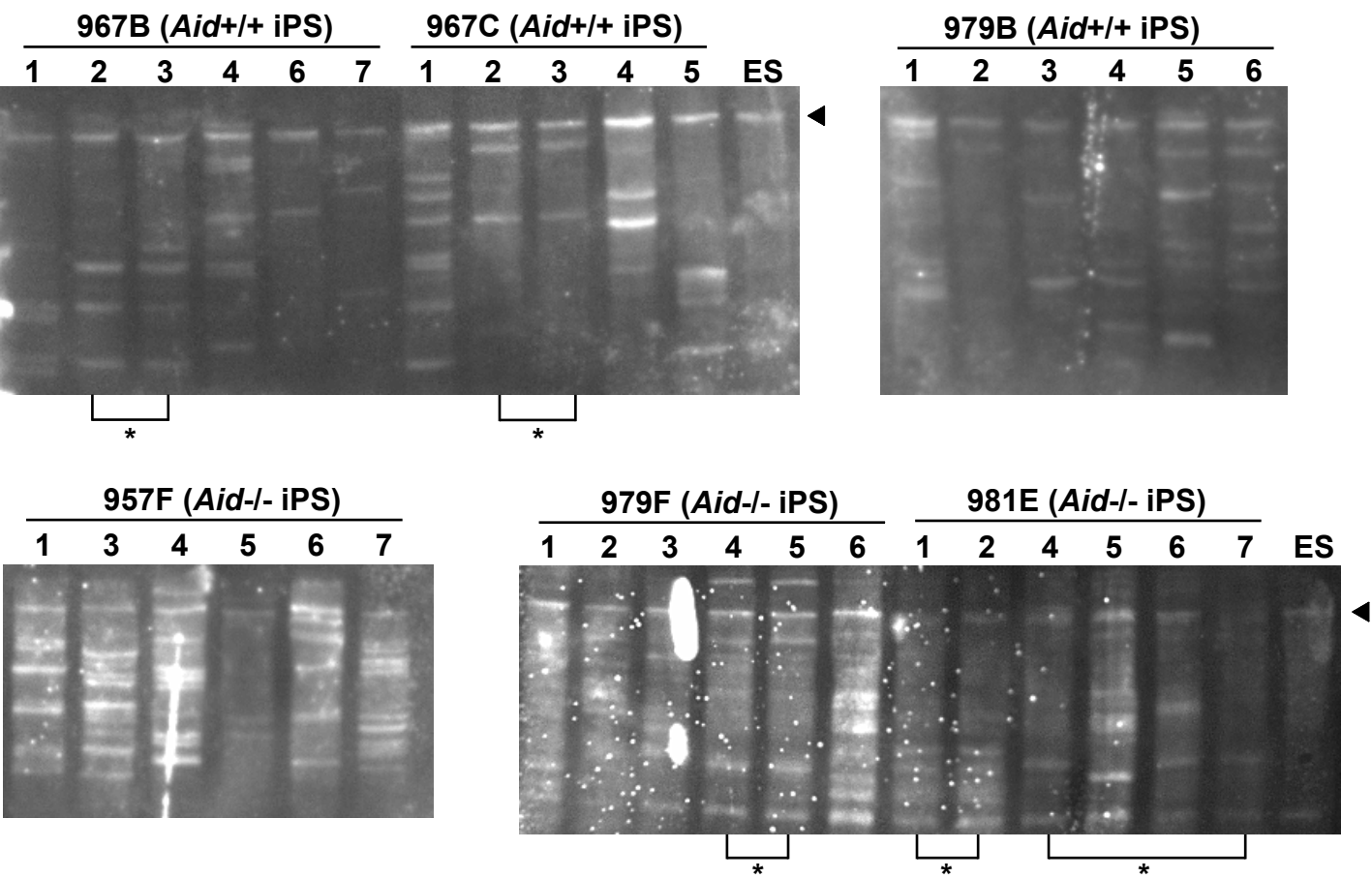

Supplement: Figure S7 — The results of a Southern blot analysis of the transgene integration with a Klf4 cDNA probe. The arrowhead indicates the endogenous Klf4 allele. Aid +/+ iPS cell clones, 967B2 and 967B3 and 967C2 and 967C3; and Aid −/− iPS cell clones, 979F4 and 979F5, 981E1 and 981E2 and 981E4 and 981E7, were apparently the same clones based on their integration patterns (asterisk). (PDF) [file pone.0094735.s007.pdf]

Figure S8

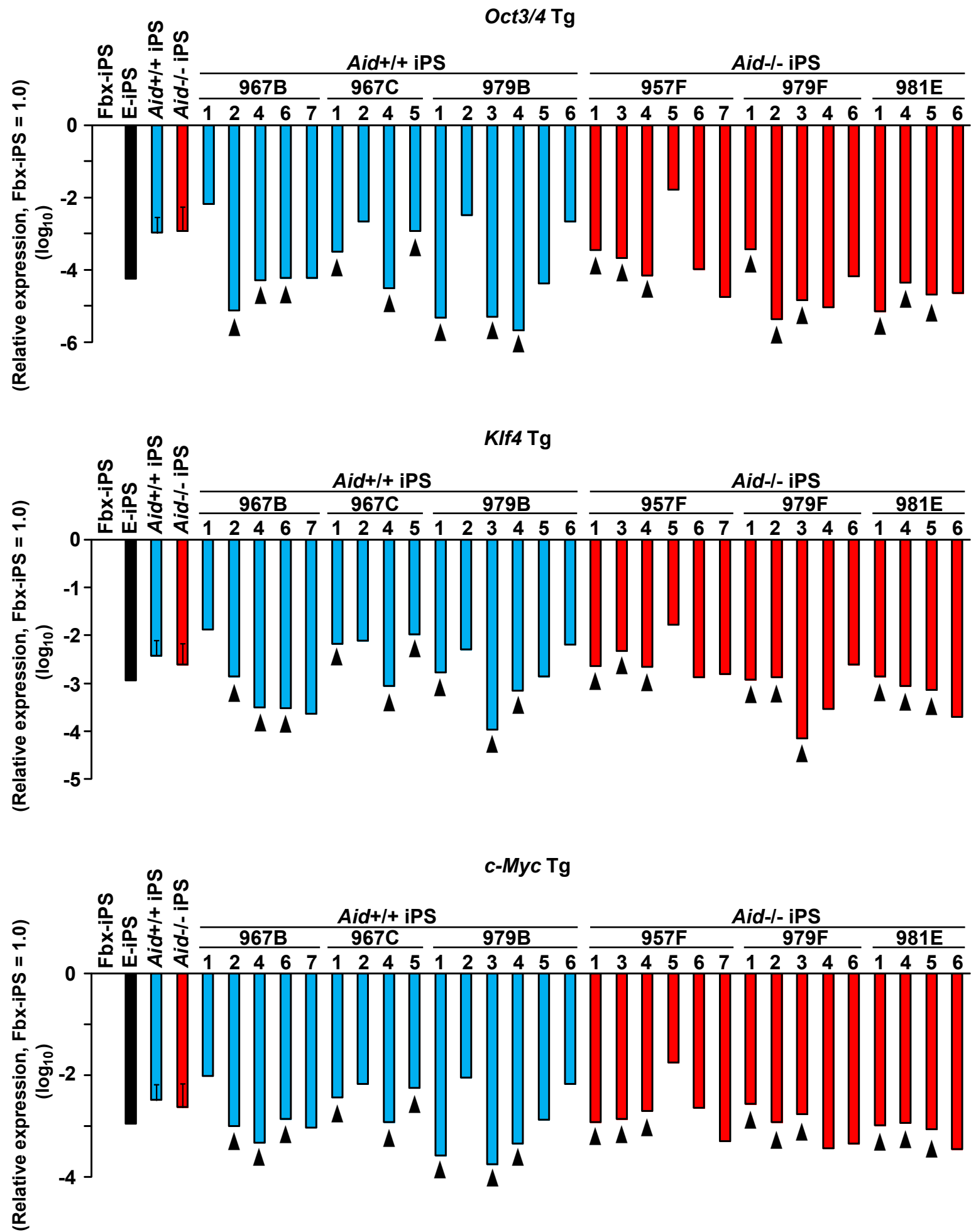

Supplement: Figure S8 — The relative expression levels of transgenes. Total RNA was isolated from Fbx15 reporter iPS cells (Fbx-iPS) [1], established iPS cells (20D17 and 178B5), Aid +/+ iPS cell clones and Aid −/− iPS cell clones, and was used for a quantitative RT-PCR analysis. Each experiment was repeated two times, and the averages are shown. The data were normalized to Gapdh, and the data for Fbx-iPS cells was set at a relative level of 1. The third and fourth bars from the left side show the averages of the Aid +/+ and Aid −/− iPS clones, respectively. The error bars represent the SD of the clones. The arrowhead indicates the clones selected for characterization. (PDF) [file pone.0094735.s008.pdf]

Figure S9

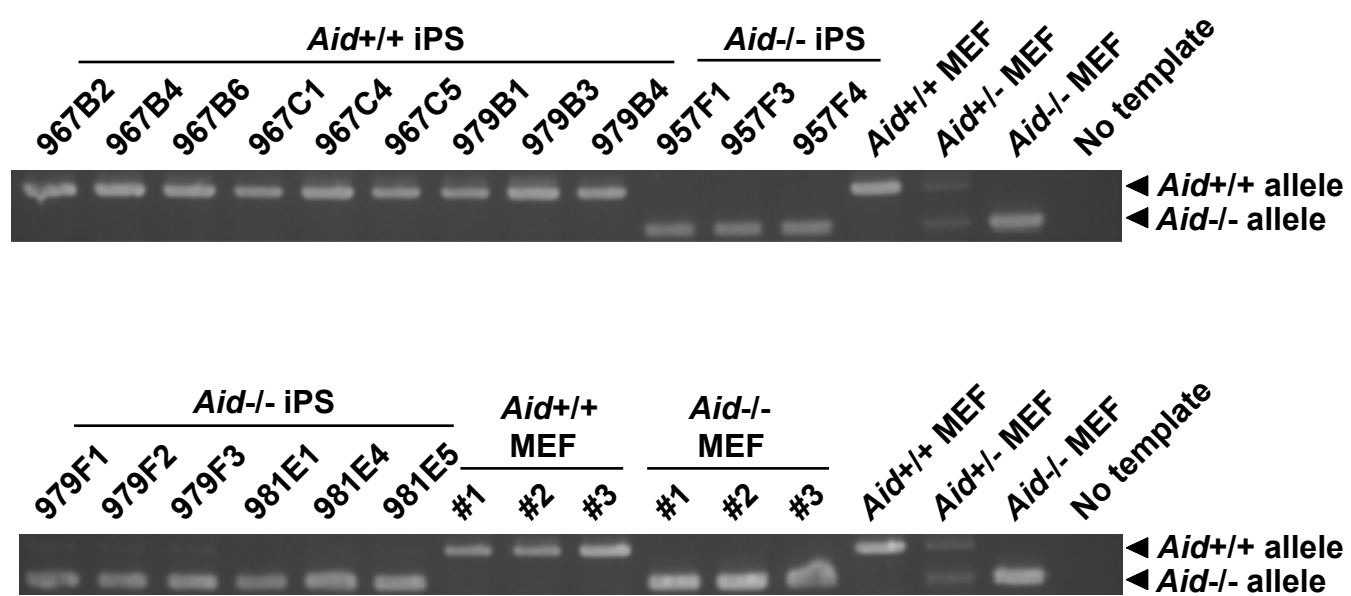

Supplement: Figure S9 — The results of a genotyping analysis of the Aid +/+ and Aid −/− iPS cell clones. Genomic DNA was isolated from Aid +/+ iPS cell clones and Aid −/− iPS cell clones. The genotyping analysis was performed by PCR. The primers used for this experiment are listed in Table S7. (PDF) [file pone.0094735.s009.pdf]

Figure S10

A

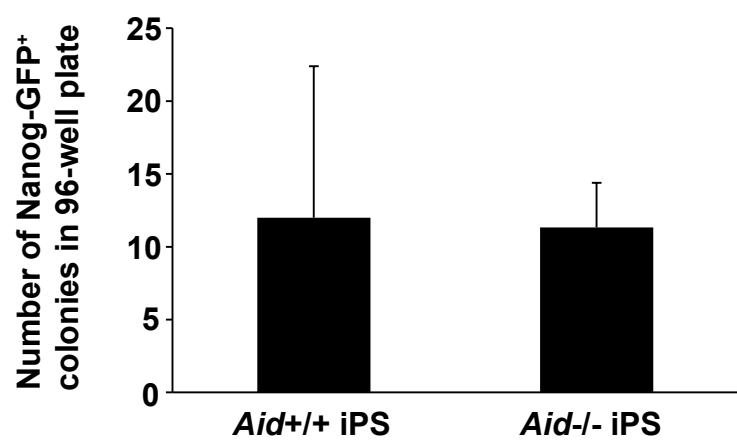

B

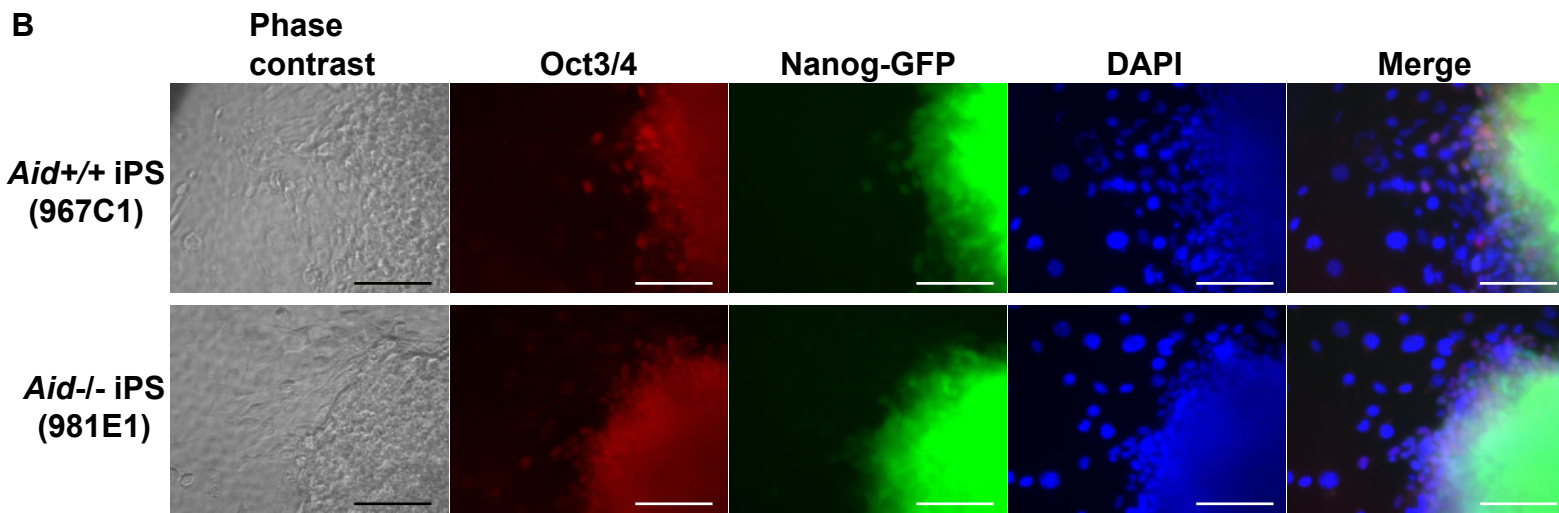

Supplement: Figure S10 — Clonogenic assay. (A) The number of Nanog-GFP positive colonies in 96-well plates. Single Aid +/+ and Aid −/− iPS cells were plated into 96-well plates, and the number of Nanog-GFP-positive colonies was counted after seven days. The data are the averages ± SD of three iPS cell clones. (B) The expression of the Oct4 and Nanog-GFP proteins. Aid +/+ and Aid −/− iPS cell colonies were stained with an antibody for Oct3/4, along with 4', 6-Diamidino-2-Phenylindole (DAPI). Bars; 100 μm. (PDF) [file pone.0094735.s010.pdf]

Figure S11

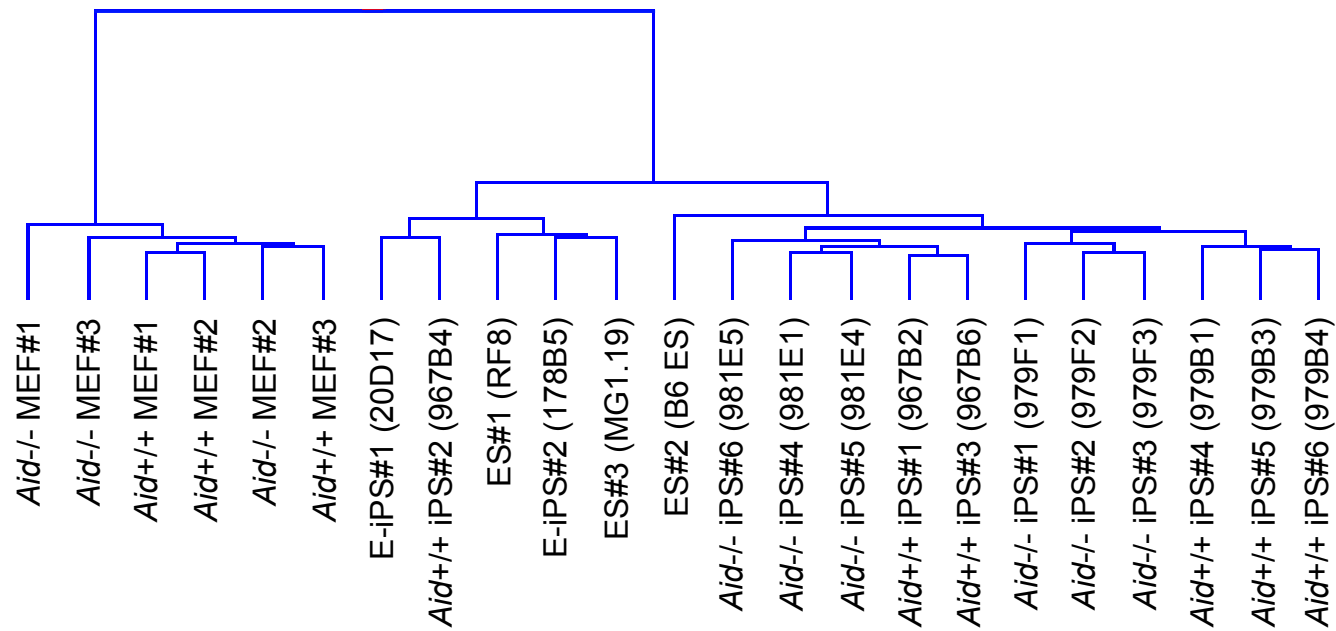

Supplement: Figure S11 — Hierarchical clustering. The hierarchical clustering analysis of gene expression was performed using all detected probes. (PDF) [file pone.0094735.s011.pdf]

Figure S12

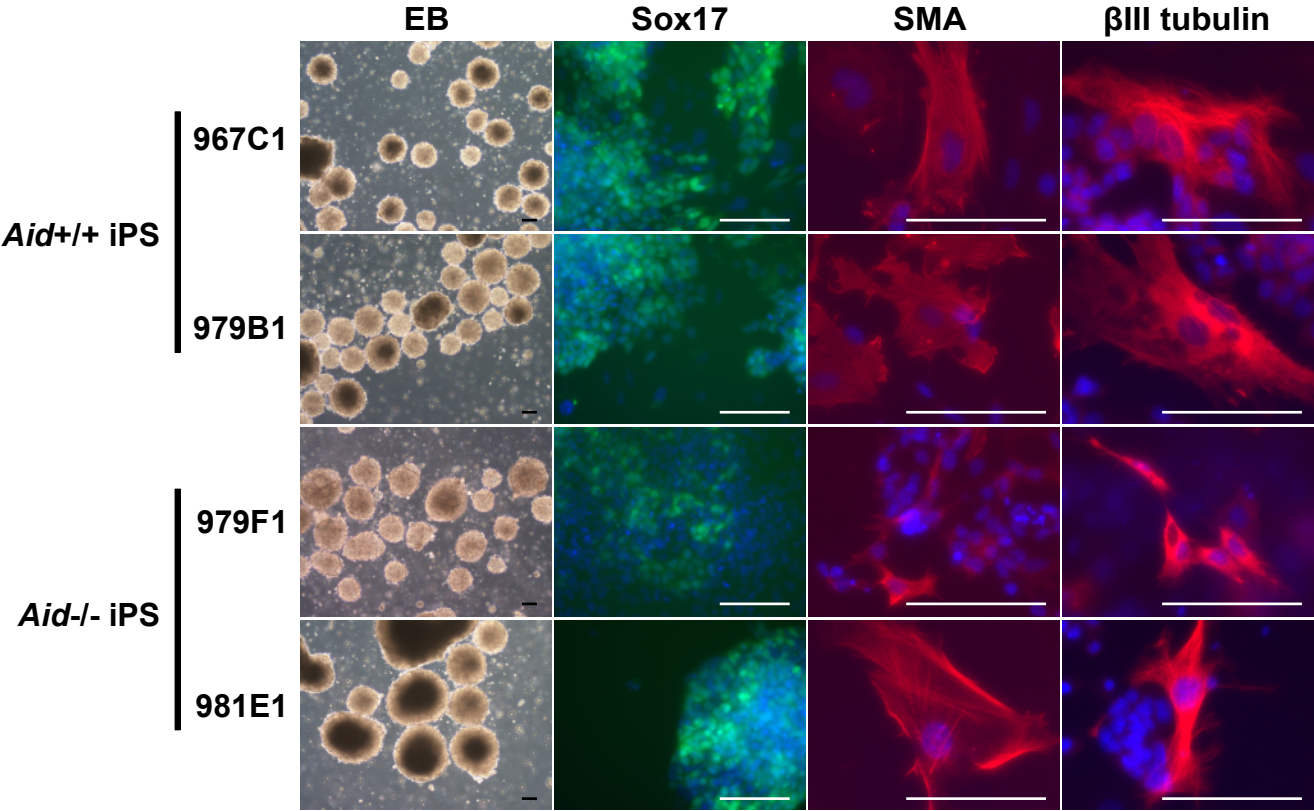

Supplement: Figure S12 — In vitro differentiation of Aid −/− iPS cells. Aid +/+ (967C1 and 979B1) and Aid −/− iPS cell clones (979F1 and 981E1) were differentiated in vitro through the formation of EBs, and were stained with antibodies for Sox17, SMA and βIII tubulin. Bars; 100 μm. (PDF) [file pone.0094735.s012.pdf]
