# Supplementary material for: Generation and Characterization of Induced Pluripotent Stem Cells from Aid-Deficient Mice
Source: PLoS One. 2014 Apr 9;9(4):e94735. doi: 10.1371/journal.pone.0094735 (PMC3981863; doi:10.1371/journal.pone.0094735)

Figure S13

A

Aid+/+ MEFs

Clone name	*Aid*+/+ MEF#1	*Aid*+/+ MEF#2	*Aid*+/+ MEF#3
Aid+/+ MEF#1		69%	62%
Aid+/+ MEF#2			53%
Aid+/+ MEF#3			

Aid+/+ iPS cells

Clone name	967B2	967B4	979B1	979B3
967B2		69%	66%	60%
967B4			78%	71%
979B1				77%
979B3				

Aid-/- iPS cells

Clone name	979F1	979F3	981E1	981E4
979F1		77%	74%	68%
979F3			74%	67%
981E1				63%
981E4				

ES cells

Clone name	RF8	MG1.1	B6 ES
RF8		44%	46%
MG1.19			27%
B6 ES			

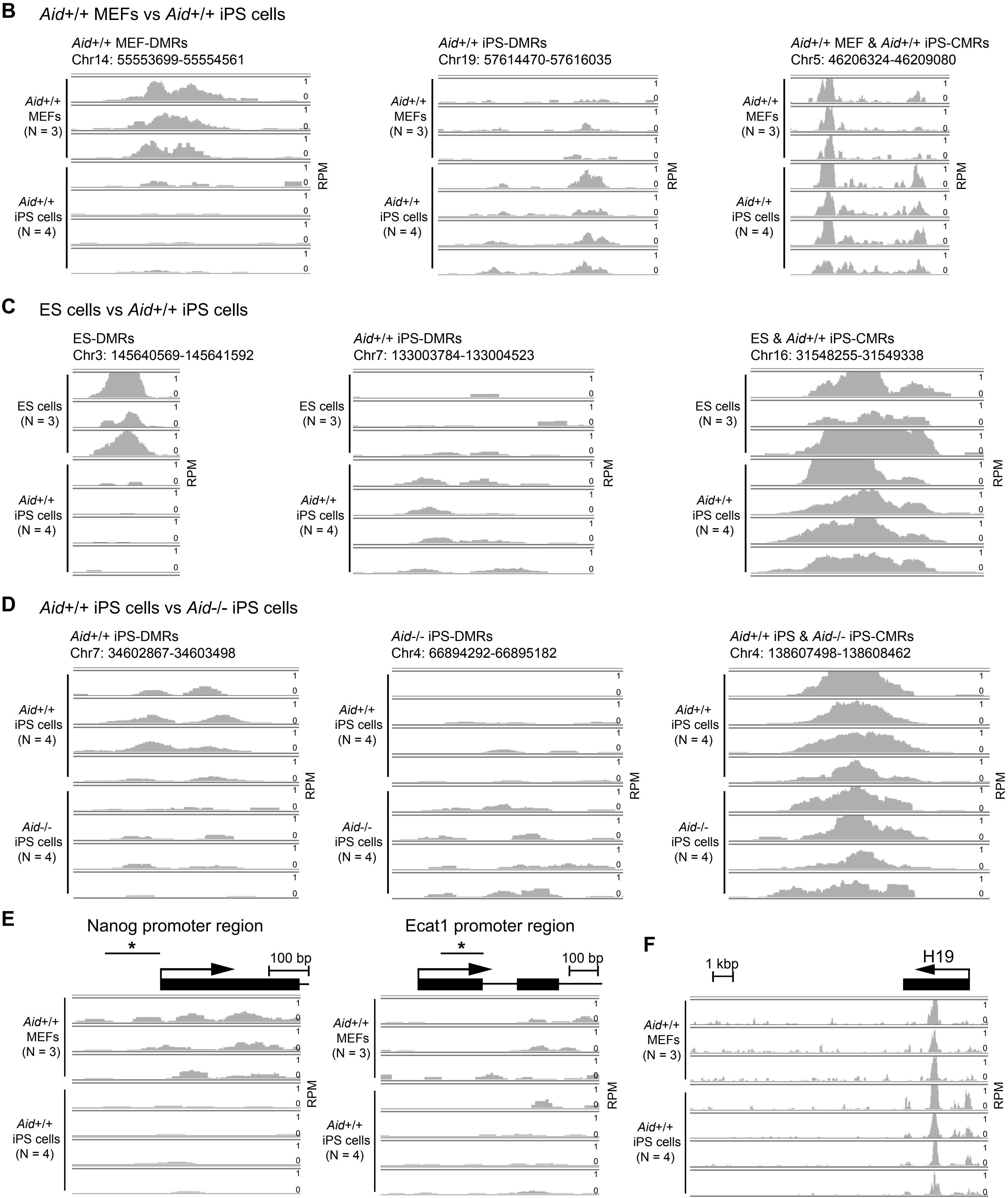

Supplement: Figure S13 — MBD-sequencing. (A) The proportion of overlapping methylated regions between biological replicates. The proportion was calculated by dividing the number of overlapping regions by the number of total regions detected in the two samples. (B–D) Representative methylated regions identified by the comparison of Aid +/+ MEFs and Aid +/+ iPS cells (B), ES cells and Aid +/+ iPS cells (C) and Aid +/+ and Aid −/− iPS cells (D). RPM; Reads per million mapped reads. (E) The number of mapped reads at the Nanog and Ecat1 promoter regions in Aid +/+ MEFs and Aid +/+ iPS cells. Asterisks indicate the regions examined by pryosequencing in Figs. 2D (Nanog promoter) and 3A (Ecat1 promoter). (F) Representative small peaks in Aid +/+ MEFs. (PDF) [file pone.0094735.s013.pdf]
