# Supplementary material for: Generation and Characterization of Induced Pluripotent Stem Cells from Aid-Deficient Mice
Source: PLoS One. 2014 Apr 9;9(4):e94735. doi: 10.1371/journal.pone.0094735 (PMC3981863; doi:10.1371/journal.pone.0094735)
Supplement: Table S1 — The efficiency of establishing Aid −/− iPS cells. (PDF) [file pone.0094735.s014.pdf]

Supplementary Table 1.

The efficiency of establishing *Aid*$^{-/-}$ iPS cells.

Experiment	Efficiency (%)	
	Aid $^{+/+}$ iPS	*Aid* $^{-/-}$ iPS
Exp. 967	72 (18/25)	75 (9/12)
Exp. 979	100 (12/12)	100 (12/12)
Exp. 980	100 (6/6)	100 (6/6)
Average	90.7 ± 16.2	91.7 ± 14.4

Nanog-GFP positive *Aid*$^{+/+}$ and *Aid*$^{-/-}$ iPS colonies were picked up mechanically and passaged four times on feeder cells and two times on gelatin coated dish. Judging by their morphology and GFP fluorescence, number of undifferentiated clones was counted. Number in parenthesis indicates number of undifferentiated clones among total clones.
