# Supplementary material for: Generation and Characterization of Induced Pluripotent Stem Cells from Aid-Deficient Mice
Source: PLoS One. 2014 Apr 9;9(4):e94735. doi: 10.1371/journal.pone.0094735 (PMC3981863; doi:10.1371/journal.pone.0094735)
Supplement: Table S2 — A list of the differentially expressed probes between Aid +/+ and Aid −/− iPS cells. (PDF) [file pone.0094735.s015.pdf]

Supplementary Table 2.

A list of the differentially expressed probes between *Aid*$^{+/+}$ and *Aid*$^{-/-}$ iPS cells.

ProbeName	GeneSymbol	Regulation
A_51_P358894	Ttc9b	up
A_55_P2107775	Apol9a	up
A_30_P01021193		up
A_51_P394814	Svep1	up
A_55_P1974612	C030039L03Rik	up
A_52_P154741	Lgals6	up
A_51_P318830	Syt10	up
A_55_P2168223	Aicda	up
A_30_P01029269		up
A_52_P560146	Gm22	up
A_30_P01024160		up
A_30_P01019832		up
A_55_P2022288	Gm6662	up
A_30_P01020677		up
A_30_P01025542		up
A_30_P01023098		up
A_30_P01030925		up
A_52_P244956	Aicda	up
A_30_P01030806		up
A_55_P2027731	Il1rl1	up
A_52_P191567	Plcl1	up
A_51_P355753	Hic1	up
A_30_P01018499		up
A_52_P117393	Tlr6	up
A_30_P01028192		up
A_30_P01031183		up
A_51_P334979	Apoc2	down
A_55_P2090768		down
A_30_P01025529		down
A_52_P148514	Hpse	down
A_51_P345340	2310043J07Rik	down
A_55_P2052071	Gsx2	down
A_55_P2196447	9430069I07Rik	down
A_30_P01029697		down
A_30_P01023023		down
A_55_P2055315		down
A_52_P150547	Cd8a	down
A_30_P01025622		down

Six clones of *Aid*$^{+/+}$ and *Aid*$^{-/-}$ iPS cells were compared with microarray (fold change > 2, corrected *p*-value < 0.05). Twenty six up-regulated and 12 down regulated probes are detected.
