# Supplementary material for: Generation and Characterization of Induced Pluripotent Stem Cells from Aid-Deficient Mice
Source: PLoS One. 2014 Apr 9;9(4):e94735. doi: 10.1371/journal.pone.0094735 (PMC3981863; doi:10.1371/journal.pone.0094735)
Supplement: Table S3 — A summary of the blastocyst injections. (PDF) [file pone.0094735.s016.pdf]

Supplementary Table 3.
A summary of the blastocyst injection.

Clone name		Number of born mice	Number of chimeras
Aid $^{-/-}$ iPS cells	946B27	101	35
	946B28	37	13
	953E15	47	13
	953E29	44	21
	1007KF1	38	7
	1007KF3	16	1
	1007KF5	11	1
	1007KF6	27	0
Aid $^{+/+}$ iPS cells	1007KA1	21	1
	1007KA2	21	5
	1007KA3	13	2

Three *Aid*$^{+/+}$ iPS cell clones and eight *Aid*$^{-/-}$ iPS cell clones were tested. Number of born mice and chimeras are shown.
