# Supplementary material for: Generation and Characterization of Induced Pluripotent Stem Cells from Aid-Deficient Mice
Source: PLoS One. 2014 Apr 9;9(4):e94735. doi: 10.1371/journal.pone.0094735 (PMC3981863; doi:10.1371/journal.pone.0094735)
Supplement: Table S4 — A summary of the MBD-seq findings. (PDF) [file pone.0094735.s017.pdf]

Supplementary Table 4.
A summary of the MBD-seq findings.

Clone name		Total reads	Number of reads mapped to reference	Ratio (%)	Number of detected regions
Aid $^{+/+}$ iPS cells	967B2	33,467,319	28,386,827	85	78,847
	967B4	41,086,765	36,374,628	89	88,332
	979B1	41,090,944	36,919,117	90	100,339
	979B3	39,983,206	35,696,402	89	110,943
Aid $^{-/-}$ iPS cells	979F1	34,063,142	27,101,491	80	91,536
	979F3	33,582,883	27,226,008	81	91,985
	981E1	39,004,447	31,281,996	80	90,683
	981E4	32,280,472	24,950,886	77	69,669
ES cells	RF8	32,061,578	20,124,759	63	58,916
	MG1.19	35,230,050	26,874,357	76	33,694
	B6 ES	41,642,948	29,714,737	71	106,385
Aid $^{+/+}$ MEFs	#1	38,856,522	27,563,519	71	48,395
	#2	37,404,390	30,113,821	81	57,893
	#3	35,428,777	27,825,300	79	35,546
