# Supplementary material for: Generation and Characterization of Induced Pluripotent Stem Cells from Aid-Deficient Mice
Source: PLoS One. 2014 Apr 9;9(4):e94735. doi: 10.1371/journal.pone.0094735 (PMC3981863; doi:10.1371/journal.pone.0094735)
Supplement: Table S5 — The classification of the methylated regions detected by MBD-seq. (PDF) [file pone.0094735.s018.pdf]

Supplementary Table 5.
The classification of the methylated regions detected by MBD-seq.

(A) *Aid*$^{+/+}$ iPS cells vs *Aid*$^{+/+}$ MEFs

	Region				
	Promoter	Terminator	Gene body	Others	Total
Aid$^{+/+}$ iPS-DMRs	372	477	7,976	8,546	17,371
Aid$^{+/+}$ MEF-DMRs	152	96	828	858	1,934
CMRs	540	796	9,887	12,930	24,153

(B) *Aid*$^{+/+}$ iPS cells vs ES cells

	Region				
	Promoter	Terminator	Gene body	Others	Total
Aid$^{+/+}$ iPS-DMRs	22	29	440	681	1,172
ES-DMRs	3	0	11	16	30
CMRs	390	645	9,392	14,525	24,952

(C) *Aid*$^{+/+}$ iPS cells vs *Aid*$^{-/-}$ iPS cells

	Region				
	Promoter	Terminator	Gene body	Others	Total
Aid$^{+/+}$ iPS-DMRs	4	3	104	123	234
Aid$^{-/-}$ iPS-DMRs	1	1	18	32	52
CMRs	1,143	1,640	22,644	26,301	51,728

Methylted regions were classified into promoter (within two kbp upstream from transcription start site), terminator (within two kbp downstream from transcription terminator), genebody (between transcription start site and transcription terminator) and others.
