# Supplementary material for: Generation and Characterization of Induced Pluripotent Stem Cells from Aid-Deficient Mice
Source: PLoS One. 2014 Apr 9;9(4):e94735. doi: 10.1371/journal.pone.0094735 (PMC3981863; doi:10.1371/journal.pone.0094735)
Supplement: Table S7 — The primers used in the present study. (PDF) [file pone.0094735.s020.pdf]

Supplementary Table 7.
The primers used in the present study.

Name	Sequence	Applications	Reference
mAid F	CACCATGGACAGCCTTCTGATGAA	Cloning/Aid	
mAid R	TCAAAATCCCAACATACGAAATGC A		
mApobec1 F	CACCATGAGTTCCGAGACAGGCCC	Cloning/Apobec1	
mApobec1 R	TCATTTCAACCCTGTAGCCCAAAG G		
mApobec1 H61K_S	GAAGTTGACTTCAACTTTGTTGCTGGTGTTTTG	Mutagenesis/Apobec1	[20]
mApobec1 H61K_AS	CAAAACACCAGCAACAAAGTTGAAGTCAACTTC		
mApobec C93S/C96S_S	GCCCTGGAGCTCTCCCCGCTGGGACTCCAG		
mApobec C93S/C96S_AS	CTGGAGTCCCAGCGGGGAGAGCTCCAGGGC		
mKLF4cDNAs1064	CACCATGGACCCGGGCGTGGCTGCCAGAAA	cDNA probe for Souther blot analysis	[1]
mKLF4cDNAas1769	TTAGGCTGTTCTTTTCCGGGGCCACGA		
Control PrimerF	ACCACAGTCCATGCCATCAC	PCR/Gapdh	
Control Primer R	TCCACCACCCTGTTGCTGTA		
Oct3/4-S9	TCTTTCCACCAGGCCCCCGGCTC	PCR/Oct3/4	
Oct3/4-AS210	TGCGGGCGGACATGGGGAGATCC		
6047-S4	AGGGTCTGCTACTGAGATGCTCTG	PCR/Nanog	
6047-AS5	CAACCACTGGTTTTTCTGCCACCG		
45328-S118	ACTGCCCCTCATCAGACTGCTACT	PCR/ERas	
ERas-AS304	CACTGCCTTGTACTCGGGTAGCTG		
pH34-U38	GAAGTCTGGTTCCTTGGCAGGATG	PCR/Esg1	
pH34-L394	ACTCGATACACTGGCCTAGC		
Ecat1-RT-S	TGTGGGGCCCTGAAAGGCGAGCTGAGA T	PCR/Ecat1	
Ecat1-RT-AS	ATGGGCCGCCATACGACGACGCTCAACT		
mOCt3/4-S1120	CCCTGGGGATGCTGTGAGCCAAGG	PCR/Oct3/4 Tg	
pMXs-AS3200	TTATCGTCGACCACTGTGCTGCTG		
Sox2-S768	GGTTACCTCTTCCTCCCACTCCAG	PCR/Sox2 Tg	
pMXs-AS3200	TTATCGTCGACCACTGTGCTGCTG		
Klf4-S1236	GCGAACTCACACAGGCGAGAAACC	PCR/Klf4 Tg	
pMXs-AS3200	TTA TCG TCG ACC ACT GTG CTG CTG		
c-Myc-S1093	CAG AGG AGG AAC GAG CTG AAG CGC	PCR/c-Myc Tg	
pMXs-AS3200	TTATCGTCGACCACTGTGCTGCTG		
mAID-185-S	CGTGGTGAAGAGGAGAGATAGTG	PCR/Aid	
mAID-295-AS	CAGTCTGAGATGTAGCGTAGGAA		
Apobec1 Fw3	GAGCCGACACCCCTATGTAA	PCR/Apobec1	
Apobec1 Rv3	TTGGCCAATAAGCTTCGTTTGA		
mAID-238-S	CTGCCAAACCTGATGTCTTGAGTTTGAT	Genotyping PCR/*Aid* $^{-/-}$ allele	[12]
mAID-L3-AS	AACCAAGCCTATGCCTACAGCATCCAGG		
mAID-227-S	CAACGTGGCGTCCAAACAGGCACTTCCG	Genotyping PCR/*Aid* $^{+/+}$ allele	
mAID-175-AS	GGTCCCAGTCTGAGATGTAGCGTAGG		
pLKO.1-shAid#1-sense	CCGGGAAGTCGATGACTTGCGAGATCTCGAGATCTCGCAAGTCATCGACTTC TTTTTG	shAid#1	[19]
pLKO.1-shAid#1-antisense	AATTCAAAAAGAAGTCGATGACTTGCGAGATCTCGAGATCTCGCAAGTCATCGACTT C		
pLKO.1-shAid#2-sense	CCGGCATGACCTTCAAAGACTATTTCTCGAGAAATAGTCTTTGAAGGTCATGTTTTTG	shAid#2	
pLKO.1-shAid#2-antisense	AATTCAAAAACATGACCTTCAAAGACTATTTCTCGAGAAATAGTCTTTGAAGGTCATG		
pLKO.1-shAid#3-sense	CCGGCTGTGACTTAGAAACTTCTCTCGAGAGAAGTTTCTAAGTCACAGTTTTTG	shAid#3	
pLKO.1-shAid#3-antisense	AATTCAAAAACTGTGACTTAGAAACTTCTCTCGAGAGAAGTTTCTAAGTCACAG		
pLKO.1-shAid#4-sense	CCGGCAGTCGCCATTATAATGCACTCGAGTGCATTATAATGGCGACTG TTTTTG	shAid#4	
pLKO.1-shAid#4-antisense	AATTCAAAAACAGTCGCCATTATAATGCACTCGAGTGCATTATAATGGCGACTG		
meNanog-F2-S	GATTTTGTAGGTGGGATTAATTGTGAATTT	Pyrosequencing/Nanog-PCR	[*]
meNanog-333AS-5'bio	Bio-ACTAAATTCCTTACCAACCTCTATAC		
mNanog region8-p1,2-pyroseq	GAATTTATAGGGTTGGTG	Pyrosequencing/Nanog-Sequence	
mNanog region8-p3,4-pyroseq	AGGAGTAGGATTTATTTTTTAAATT		
Oct PRO F	TGGGTTGAAATATTGGGTTTATTT	Pyrosequencing/Oct3/4-PCR	
Oct PRO R-bio	Bio-CTAAAACCAAATATCCAACCATA		
mOct4 region2-p4,5,6-pyroseq	GTAGTGTTAATAGGTTTTGT	Pyrosequencing/Oct3/4-Sequence	
mOct4 region2-p7,8-pyroseq	GTTTAGTTTTAAGGGTTGTTTTG		
meECAT1-F1-S	TTAATGTTGGGATTAAAGGTGTGTGTTGTT	Pyrosequencing/Ecat1-PCR	
meECAT1-F1-AS-bio	Bio-TTACCTTAACCTTTCCAATTCACTTACCAA		
Ecat1prom1-119CpG#1-4 seq	GATTTTGAGTTTGTATAGGT	Pyrosequencing/Ecat1-Sequence	
Ecat1prom1-119CpG#5-7 seq	TTTAAGTTGTTTAAGTGGTTTT		

*; Primers for the pyrosequence were modified from the primers used in the journal; Imamura M, Miura K, Iwabuchi K, Ichisaka T, Nakagawa M, et al. (2006) Transcriptional repression and DNA hypermethylation of a small set of ES cell marker genes in male germline stem cells. BMC developmental biology 6: 34. doi:10.1186/1471-213X-6-34.
